# Supplementary figures and images for: Graphlet correlation distance to compare small graphs
Source: PLoS One. 2023 Feb 15;18(2):e0281646. doi: 10.1371/journal.pone.0281646 (PMC9931116; doi:10.1371/journal.pone.0281646)

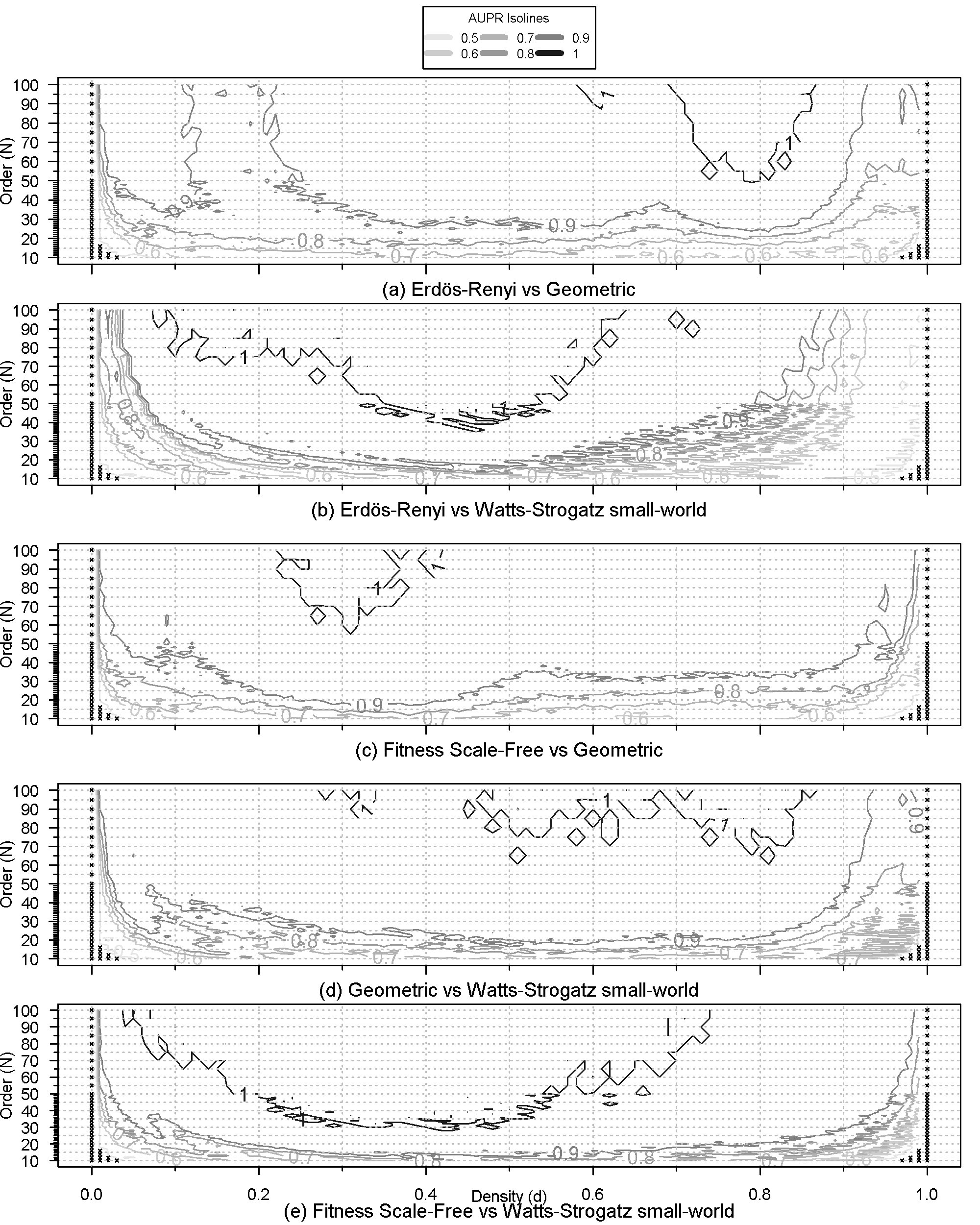

Supplement: S1 Fig — Diagram of the matrix of AUPR (AM1,M2) (a) Erdős-Rényi vs Geometric (b) Erdős-Rényi vs Watts-Strogatz small-world (c) Fitness Scale-Free vs Geometric (d) Geometric vs Watts-Strogatz small-world (e) Fitness Scale-Free vs Watts-Strogatz small-world. For each pair of models, and for each order (from 10 to 100) and edge density (from 0 to 1) combination, the quality of clustering between 100 graphs of each of the two types of models is assessed by the Area Under the Precision-Recall curve (AUPR). A maximum value of 1 corresponds to perfect discrimination. Black crosses represent zero AUPR. (TIF) [file pone.0281646.s001.tif]

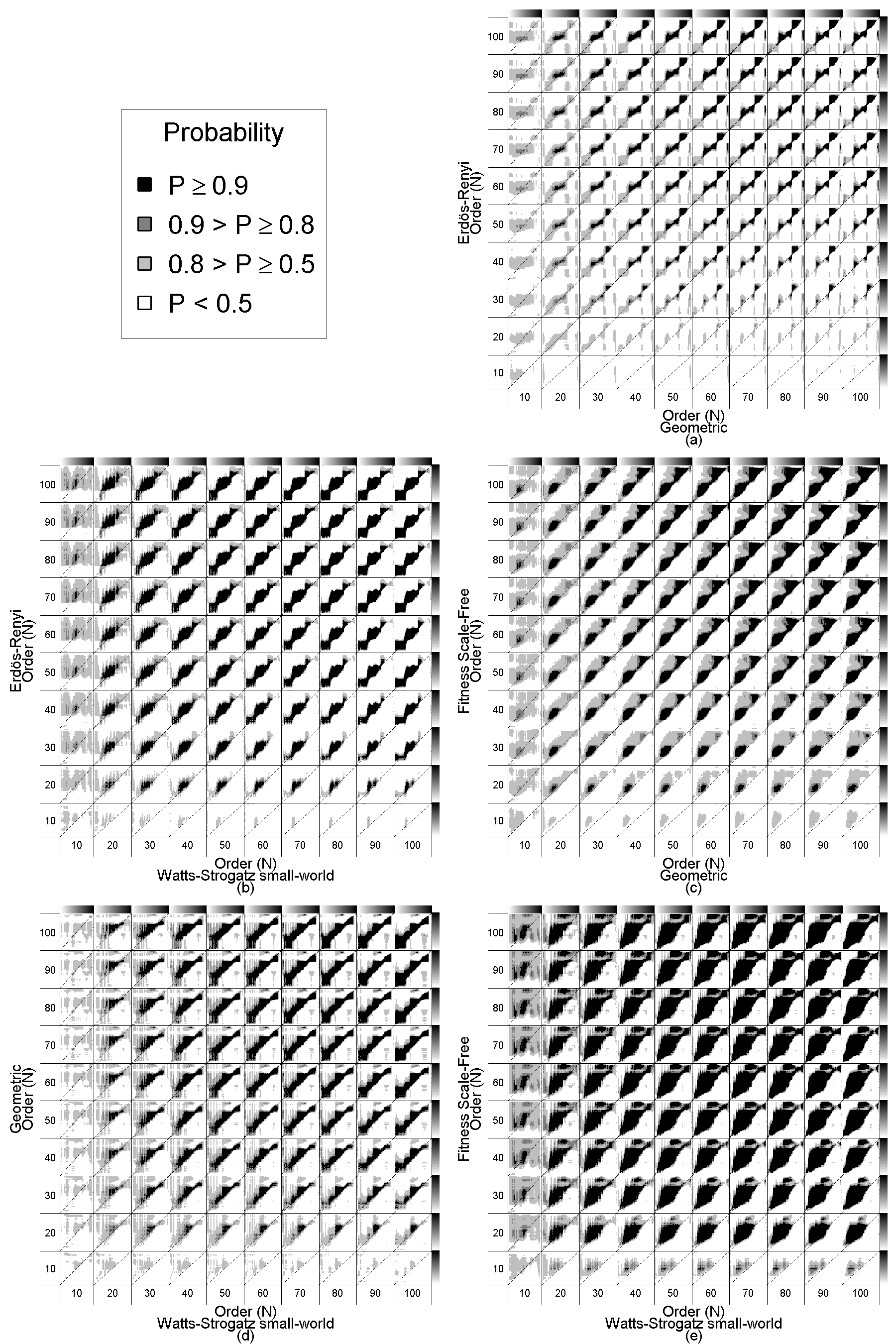

Supplement: S2 Fig — with different order and/or edge density. Each block (i, j) concerns the comparison of an M1 of order N and a M2 of order N’, with edge density d and d’ respectively ranging from 0 to 1 (grey gradient from white to black on the top and right side) with (M1, M2)∈{ER, SF, SW, GO}2 and M1 ≠ M2. Dashed lines in each block highlight comparison when d = d’. (a) Probability matrix BM1,M2 that DM1(N,d),M2(N′,d′)>max(DM1(N,d),M1(N′,d′),DM2(N,d),M2(N′,d′)) with M1 = SF and M2 = GO. (b) BER, SW, (c) BSF, GO, (d) BGO, SW, (e) BSF, SW. (TIF) [file pone.0281646.s002.tif]
