# Supplementary material for: Graphlet correlation distance to compare small graphs
Source: PLoS One. 2023 Feb 15;18(2):e0281646. doi: 10.1371/journal.pone.0281646 (PMC9931116; doi:10.1371/journal.pone.0281646)
Supplement: S1 Eq — We built the test by computing η the number of times the standardised distance between GCMG and GCM¯M is smaller or equal to the distance δstd,Mk. The p-value [50] is defined by p^=(η+1)/(K+1). The larger the p-value, the less evidence against H0. (PDF) [file pone.0281646.s003.pdf]

S1 Eq To account for the difference in variability between the correlation coefficients of each pair of orbits, we also computed the following standardised distance δ_{std,M_k} between $GCM(M_k)$ and $\overline{GCM}_M$ where $\sigma(i,j)$ is the standard deviation of the correlation coefficients of the pair of orbits (i, j) under H_0. We built the test by computing η the number of times the standardised distance between GCM_G and $\overline{GCM}_M$ is smaller or equal to the distance δ_{std,M_k}. The p-value [50] is defined by $\hat{p} = (\eta + 1)/(K + 1)$. The larger the p-value, the less evidence against H_0.

$$\delta_{std,M_k} = \sqrt{\sum_{i=1}^{11} \sum_{j=i+1}^{11} \left(\frac{\overline{GCM}_M(i,j) - GCM(M_k)(i,j)}{\sigma(i,j)} \right)^2} \tag{1}$$
