# Supplementary material for: Graphlet correlation distance to compare small graphs
Source: PLoS One. 2023 Feb 15;18(2):e0281646. doi: 10.1371/journal.pone.0281646 (PMC9931116; doi:10.1371/journal.pone.0281646)
Supplement: S1 Table — Each empirical graph is associated with an estimated p-value (p^) of being an outcome of an Erdős-Rényi, Fitness scale-free model, a Watts-Strogatz small word or a Geometric model. As in Table 1, empirical graphs are sorted according to their order. (p^*<0.05, p^**<0.01 and p^***≤0.001) (PDF) [file pone.0281646.s004.pdf]

S1 Table Estimated p-values (std). Each empirical graph is associated with an estimated p-value ($\hat{p}$) of being an outcome of an Erdős-Rényi, Fitness scale-free model, a Watts-Strogatz small word or a Geometric model. As in Table 1, empirical graphs are sorted according to their order. ($\hat{p}^{*} < 0.05$, $\hat{p}^{**} < 0.01$ and $\hat{p}^{***} \leq 0.001$)

	**Standardised *p*-value**			
**Graph**	**Erdős-Rényi**	**Scale-Free**	**Watts-Strogatz**	**Geometric**
Graph 1	0.001***	0.001***	0.001***	0.095
Graph 2	0.001***	0.001***	0.001***	0.160
Graph 3	0.001***	0.001***	0.002**	0.092
Graph 4	0.001***	0.020*	0.001***	0.039*
Graph 5	0.001***	0.001***	0.001***	0.102
Graph 6	0.001***	0.001***	0.001***	0.001***
Graph 7	0.001***	0.001***	0.001***	0.003**
Graph 8	0.001***	0.001***	0.001***	0.004**
Graph 9	0.001***	0.001***	0.001***	0.003**
Graph 10	0.001***	0.001***	0.001***	0.173
