# Supplementary material for: Graphlet correlation distance to compare small graphs
Source: PLoS One. 2023 Feb 15;18(2):e0281646. doi: 10.1371/journal.pone.0281646 (PMC9931116; doi:10.1371/journal.pone.0281646)

PLOS ONE

Graphlet correlation distance to compare small graphs

--Manuscript Draft--

**Manuscript Number:**	PONE-D-22-11020
**Article Type:**	Research Article
**Full Title:**	Graphlet correlation distance to compare small graphs
**Short Title:**	Graphlet correlation distance to compare small graphs
**Corresponding Author:**	Jérôme ROUX, Ph.D<br>IFREMER - Centre de Nantes: Ifremer Centre Atlantique<br>NANTES, France FRANCE
**Keywords:**	graphlets; graph comparison; random graph models
**Abstract:**	Graph models are standard tools for representing mutual relationships between sets of entities. In most scientific fields, graph have been used to study the organisation of large group of entities with a small number of connections (e.g. social media relationships, infectious disease spread). A few years ago, the Graphlet Correlation Distance (GCD) was proposed as a graph distance to assess similarity between graphs. This paper deals with two main gaps in the literature.<br>First, we assess the performance of GCD using a numerical experimental design to extend its domain of applicability in the small graph domain characterised by small numbers of entities and high densities of connections. We study its discriminating power with respect to the density and order of the graphs, but also with respect to the differences in order and density between the compared graphs. Second, we develop a statistical test based on the GCD to compare empirical graphs to three possible null models (Erd˝os-R´enyi, Barb´asi-Albert scale free and k-regular) for both small and large-size graphs. Finally, we illustrate the relevance of this approach by using two fishing case studies to assess the independence of observed proximities between fishing vessels modeled by graphs. The statistical test does not rule out independent behavior within one of the two fleets studied.
**Order of Authors:**	Jérôme ROUX
	Nicolas Bez
	Stéphanie Mahévas
	Paul Rochet
	Rocío Joo
**Additional Information:**	
**Question**	**Response**
**Financial Disclosure**<br><br>Enter a financial disclosure statement that describes the sources of funding for the work included in this submission. Review the submission guidelines for detailed requirements. View published research articles from *PLOS ONE* for specific examples.<br><br>This statement is required for submission and **will appear in the published article** if the submission is accepted. Please make sure it is accurate.	JR, Conseil Regional Pays de la Loire.<br>SM, National research project TRACFLO IFREMER.<br>The funders had no role in study design, data collection and analysis, decision to publish, or preparation of the manuscript.

Powered by Editorial Manager® and ProduXion Manager® from Aries Systems Corporation

**Unfunded studies**<br>Enter: *The author(s) received no specific funding for this work.*<br><br>**Funded studies**<br>Enter a statement with the following details:<br>• Initials of the authors who received each award<br>• Grant numbers awarded to each author<br>• The full name of each funder<br>• URL of each funder website<br>• Did the sponsors or funders play any role in the study design, data collection and analysis, decision to publish, or preparation of the manuscript?<br>• **NO** - Include this sentence at the end of your statement: *The funders had no role in study design, data collection and analysis, decision to publish, or preparation of the manuscript.*<br>• **YES** - Specify the role(s) played.<br><br>* typeset	
**Competing Interests**<br><br>Use the instructions below to enter a competing interest statement for this submission. On behalf of all authors, disclose any competing interests that could be perceived to bias this work—acknowledging all financial support and any other relevant financial or non-financial competing interests.<br><br>This statement is required for submission and **will appear in the published article** if the submission is accepted. Please make sure it is accurate and that any funding sources listed in your Funding Information later in the submission form are also declared in your Financial Disclosure statement.<br><br>View published research articles from *PLOS ONE* for specific examples.	The authors have declared that no competing interests exist.

**NO authors have competing interests**<br><br>Enter: *The authors have declared that no competing interests exist.*<br><br>**Authors with competing interests**<br><br>Enter competing interest details beginning with this statement:<br><br>*I have read the journal's policy and the authors of this manuscript have the following competing interests: [insert competing interests here]*<br><br>* typeset	
**Ethics Statement**<br><br>Enter an ethics statement for this submission. This statement is required if the study involved:<br><br>• Human participants<br>• Human specimens or tissue<br>• Vertebrate animals or cephalopods<br>• Vertebrate embryos or tissues<br>• Field research<br><br>Write "N/A" if the submission does not require an ethics statement.<br><br>General guidance is provided below. Consult the submission guidelines for detailed instructions. **Make sure that all information entered here is included in the Methods section of the manuscript.**	N/A

Powered by Editorial Manager® and ProduXion Manager® from Aries Systems Corporation

**Format for specific study types**<br><br>**Human Subject Research (involving human participants and/or tissue)**<br>• Give the name of the institutional review board or ethics committee that approved the study<br>• Include the approval number and/or a statement indicating approval of this research<br>• Indicate the form of consent obtained (written/oral) or the reason that consent was not obtained (e.g. the data were analyzed anonymously)<br><br>**Animal Research (involving vertebrate animals, embryos or tissues)**<br>• Provide the name of the Institutional Animal Care and Use Committee (IACUC) or other relevant ethics board that reviewed the study protocol, and indicate whether they approved this research or granted a formal waiver of ethical approval<br>• Include an approval number if one was obtained<br>• If the study involved *non-human primates*, add *additional details* about animal welfare and steps taken to ameliorate suffering<br>• If anesthesia, euthanasia, or any kind of animal sacrifice is part of the study, include briefly which substances and/or methods were applied<br><br>**Field Research**<br><br>Include the following details if this study involves the collection of plant, animal, or other materials from a natural setting:<br>• Field permit number<br>• Name of the institution or relevant body that granted permission	
**Data Availability**<br><br>Authors are required to make all data underlying the findings described fully available, without restriction, and from the time of publication. PLOS allows rare exceptions to address legal and ethical concerns. See the PLOS Data Policy and FAQ for detailed information.	Yes - all data are fully available without restriction

Powered by Editorial Manager® and ProduXion Manager® from Aries Systems Corporation

A Data Availability Statement describing where the data can be found is required at submission. Your answers to this question constitute the Data Availability Statement and **will be published in the article**, if accepted.<br><br>**Important:** Stating 'data available on request from the author' is not sufficient. If your data are only available upon request, select 'No' for the first question and explain your exceptional situation in the text box.<br><br>Do the authors confirm that all data underlying the findings described in their manuscript are fully available without restriction?	
**Describe where the data may be found in full sentences. If you are copying our sample text, replace any instances of XXX with the appropriate details.**<br><br>• If the data are **held or will be held in a public repository**, include URLs, accession numbers or DOIs. If this information will only be available after acceptance, indicate this by ticking the box below. For example: *All XXX files are available from the XXX database (accession number(s) XXX, XXX.*).<br>• If the data are all contained **within the manuscript and/or Supporting Information files**, enter the following: *All relevant data are within the manuscript and its Supporting Information files.*<br>• If neither of these applies but you are able to provide **details of access elsewhere**, with or without limitations, please do so. For example:<br><br>*Data cannot be shared publicly because of [XXX]. Data are available from the XXX Institutional Data Access / Ethics Committee (contact via XXX) for researchers who meet the criteria for access to confidential data.*<br><br>*The data underlying the results presented in the study are available from (include the name of the third party*	The datasets generated during and/or analysed during the current study are available in the Jérôme ROUX GitLab repository, (https://gitlab.com/jerome-roux/project_small_graphs_comparison).

and contact information or URL).<br>• This text is appropriate if the data are owned by a third party and authors do not have permission to share the data.<br><br>* typeset	
Additional data availability information:	Tick here if the URLs/accession numbers/DOIs will be available only after acceptance of the manuscript for publication so that we can ensure their inclusion before publication.

Manuscript
Click here to access/download;Manuscript;Manuscripts.pdf

Graphlet correlation distance to compare small graphs

Jérôme Roux[1*], Nicolas Bez[2], Paul Rochet[3], Rocío Joo[4], Stéphanie Mahévas[1]

1 UMR DECOD, IFREMER, BP 21105, 44311 Nantes Cedex 03, France
2 MARBEC, IRD, Univ Montpellier, Ifremer, CNRS, INRAE, Sète, France
3 ENAC, 7 av Edouaud Belin, Toulouse, France
4 Global Fishing Watch, Washington, DC 20036, USA

* jerome.th.roux@hotmail.com

Abstract

Graph models are standard tools for representing mutual relationships between sets of entities. In most scientific fields, graph have been used to study the organisation of large group of entities with a small number of connections (e.g. social media relationships, infectious disease spread). A few years ago, the Graphlet Correlation Distance (GCD) was proposed as a graph distance to assess similarity between graphs. This paper deals with two main gaps in the literature. First, we assess the performance of GCD using a numerical experimental design to extend its domain of applicability in the small graph domain characterised by small numbers of entities and high densities of connections. We study its discriminating power with respect to the density and order of the graphs, but also with respect to the differences in order and density between the compared graphs. Second, we develop a statistical test based on the GCD to compare empirical graphs to three possible null models (Erdős-Rényi, Barbási-Albert scale free and k-regular) for both small and large-size graphs. Finally, we illustrate the relevance of this approach by using two fishing case studies to assess the independence of observed proximities between fishing vessels modeled by graphs. The statistical test does not rule out independent behavior within one of the two fleets studied.

Introduction

In ecology, the science of biological interactions, understanding the functioning of a group of individuals, be it a group of humans, animals, cells, etc, requires understanding the interactions between them [1]. For many years now, graphs and graph theory have been used to describe and study the organisation of groups of individuals [2, 3]. The simplest graphs allow to represent the presence of interactions within a group of individuals. The interactions are then, graphically, the edges between the nodes of the graph (one node = one individual). Mathematically, a graph is formalised by an adjacency matrix [4], with a number of columns and rows equal to the number of individuals, and elements taking a value equal to 1 if there is an interaction between the individuals and 0 otherwise. While such graphs are simplistic representation of relational structure, they can provide an essential and formal representation of various complex phenomena from diverse scientific fields such as protein-protein interaction [5] in biology or the interaction between social animals [6] in ecology. Comparing graphs can therefore allow us to compare groups with respect to the interactions they exhibit. There is an abundant literature in graph theory aimed at comparing graphs [7–10]. This comparison is often done in a descriptive and qualitative way by comparing synthetic indicators of graph structures [11]. For

example, by comparing the distribution of the number of links that each individual has (degree distribution [12]) or the occurrences of certain forms of links between bundles of individuals (motif distribution [13]). These descriptive approaches were first performed in domains such as sociology [14], chemistry [15] and physics in the 90's, and more recently in neuroscience to compare brain graphs [16], in genomics to compare molecular graphs from different species [17] and in behavioral ecology [18–22].

The shift to quantitative graph comparisons with the introduction of similarity or distance measures is more recent [23] and has resulted in the development of plenty of distances (see [9] for a recent review). Amongst these, the Graphlet Correlation Distance (GCD) was shown to not only outperform the others but also to be robust to order (number [illegible]des) and density differences between the graphs compared [24,25]. Graphlets are small and connected subgraphs [26,27] that extend the concept of motifs [13,28] of a graph and emerged as an accurate mining tool to provide topological information that is not exclusively local [29]. Graphlets generalize the degree distribution of a graph to the distribution of subgraphs connected to a [illegible] which is assigned a particular role (orbit) [8,30]. Yaveroğlu et al [25] showed that eleven orbits were sufficient to exhaustively describe a graph, so that the topology [11] of the graph, i.e the configuration by which the individuals of a graph are connected, can be summarized by the correlation matrix between these eleven vectors of orbits' degrees, also called the Graphlet Correlation Matrix (GCM) [25]. The GCD between two graphs is defined as the Euclidean distance between the GCM of the graphs [25].

To go beyond the comparison of simple descriptors of interactions between individuals, it is appealing to test functional hypotheses about these interactions [23]. One possible approach is to test whether a graph can be considered as an outcome of a specific random graph (null model). For example, Erdös-Rényi [31] is a graph model where the links between individuals are mutually independent. It can therefore be used as a model-null to test the absence of correlation between the interactions of individuals. Some studies based on different graph comparison methods identified the similarities between empirical graphs and the outcomes of some random graph models [30,32]. However, to the best of our knowledge, none of these approaches exploits the strong potential of GCD.

Most of the studies available in the literature focus on graphs with large number of nodes (several hundreds or thousands) and very low edge densities (≤ 0.1) [33]. However, these are not the only real-world graphs. In sociology, for example, the classical examples of Zachary's (1997) karate club network [34] and Sampson's (1968) monks' network [35] contain 34 and 18 nodes respectively. In ecology, food webs can be studied at the level of trophic groups rather than at the level of species or individuals [36] with a number of entities from 25 to 172. In fisheries, fleets may consist of only ten or a few dozen interacting actors [37]. Thus, there are multiple cases of small-size graphs applications that deserve dedicated methodological developments.

This paper deals with two main gaps in the literature. First, we assess the performance of GCD in the small graph domain to extend its domain of applicability. Second, we develop a statistical test based on the GCD to compare empirical graphs to three possible null models for both small and large-size graphs. In the first part of this paper, we present the method to assess the ability of GCD to correctly distinguish small simulated graphs from known model types (Erdős-Rényi [31], Barbási-Albert scale free [38] and k-regular [39]) by a clustering approach [25,40] using a numerical experimental design. In these numerical experiments, the orders of the graph fluctuate from 5 to 50 to mimic the range encountered in some real small graphs, while the density is completely covered from 0 to 1. We specifically address the problem of the family of k-regular graphs which are difficult graphs to solve with the GCD. We study its discriminating power with respect to the density and order of the graphs, but also with respect to the differences in order and density between the compared graphs. We then propose a statistical test based

on the GCD to evaluate whether an empirical graph can be considered as an outcome of a particular random graph. Finally, we illustrate the relevance of this approach by using two fishing case studies to assess the independence of observed proximities between fishing vessels modeled by graphs. The statistical test does not rule out independent behavior within one of the two studied fleets.

Materials and methods

Graphlets Correlation Distance (GCD)

Yaveroğlu et al [25] recently proposed to compare graphs on the basis of the first eleven non-redundant orbits graphlets of up to 4-nodes. Considering a graph G of order N, they first consider the $N \times 11$ matrix which contains for each node their orbits' degree i.e the number of times the node is presented in each of the eleven orbits. Columns are called Graphlets Degree Distribution (GDD) [30] and the first column is the standard vector of degree values. Then, the Spearman's Correlation coefficient [41] is computed between all columns of the GDD matrix to build an 11×11 matrix called the Graphlet Correlation Matrix (GCM). In this framework, the topology of a given graph G is summarised by its Graphlet Correlation Matrix denoted $\mathrm{GCM_G}$. The $\mathrm{GCD_{11}}$ between two graphs $\mathrm{G_1}$ and $\mathrm{G_2}$ is defined as the Euclidean distance between the upper triangular parts of their respective GCM :

$$GCD_{11}(G_1, G_2) = \sqrt{\sum_{i=1}^{11} \sum_{j=i+1}^{11} \big(GCM_{G_1}(i,j) - GCM_{G_2}(i,j)\big)^2} \tag{1}$$

Qualifying $\mathrm{GCD_{11}}$ on small synthetic graphs

The performance of the $\mathrm{GCD_{11}}$ to identify similarities between small graphs is assessed with an experimental design using three different models of random graphs, namely the Erdős-Rényi (ER) [31], the Barbási-Albert scale free (SF-BA) [42] and the k-regular (REG) [39] models.

The Erdős-Rényi random model is the simplest and most common uncorrelated random graph model. An Erdős-Rényi graph ER(N, d) of order N and edge density $d = 2m/\big(N(N-1)\big)$ gets m edges that are randomly and uniformly chosen among the $\binom{N}{2}$ possible edges [31]. This simple configuration results in an uncorrelated graph i.e, with a zero assortativity [43] meaning that there is not preferential attachment among nodes. In other words, the Erdős-Rényi random model generates graphs where edges are statistically independent each other (which should not be confused with the notion of an independent set of nodes [44]).

The Barbási-Albert scale free model accounts for some preferential connectivity as observed in some real-world graphs [42]. In fact, in many graphs the node degree distribution, follows a power law who[illegible]ower γ is comprised between 2 and 3 [45]. A Barbási-Albert scale free graph SF-BA(N, d, γ) of order N can be viewed as a graph where each of the N nodes and a subset of m edges are added sequentially by an iterative process. The preferential attachment means that the more connected a node is, the more likely it is to receive new edges. This "rich-get-richer" phenomenon [38] results in a graph with particular components called hubs.

A graph REG(N, k, d) of order N is said to be k-regular if each node has a degree k, i.e, if they all have the same number of neighbours [39] Given the characteristics of fleet 1, we only considered 1-regular graphs ($k = 1$). This particular k-regular graph only allows for even orders for graphs. Because of this characteristic, the outputs of the REG(N, k, d) model are totally deterministic. For any even number, an N-nodes

1-regular graph REG(N, 1, d), contains a set of $m = \frac{N}{2}$ disconnected edges. The edge density of 1-regular graphs is thus $\mathrm{d} = 1/(N-1)$.

For each model $M \in \{ER, SF\text{-}BA, REG\}$ and for a given order N and edge density d we generate 100 graphs $G^i_M(N, d)$ with $i = 1, ..., 100$. If $M \in \{ER, SF\text{-}BA\}$ we define orders and edge densities sequences as $N = (4, 5, ..., 50)$ and $d = (0, 0.01, ..., 1)$, else if $M = \{REG\}$ we define $N = (4, 6, 8, ..., 50)$ and the resultant edge density $d = 1/(N-1)$ which corresponds to an edge density range from 0.16 to 0.02.

Comparing graphs with same order and edge density

For a given order N and a given edge density d, for each couple $(M_1, M_2) \in \{ER, SF\text{-}BA, REG\}^2$ with $M_1 \neq M_2$, we compute all the pairwise GCD_{11} between their 100 respective generated graphs to construct a 200×200 distance matrix $D = \begin{bmatrix} D_{1,1} & D_{1,2} \\ D_{2,1} & D_{2,2} \end{bmatrix}$. The discriminating power of GCD_{11} is assessed by the Area Under the Precision-Recall (AUPR) curve [40] computed on the above distance matrix D. The Precision-Recall curve is obtained by varying a distance threshold ϵ over the whole range of the computed distance value in the matrix distance D. We defined 100 regularly spaced distance thresholds from $min(D)$ to $max(D)$. For each threshold $\epsilon_k, k = 1, ..., 100$, four features are computed:

- the true positives TP, as the number of pairwise distances between graphs from the same model smaller than ϵ_k;
- the true negatives TN, as the number of pairwise distances between graphs from two different models greater or equal to ϵ_k;
- the false negatives FN, as the number of pairwise distances between graphs from the same model greater or equal to ϵ_k;
- and the false positives, FP, as the number of pairwise distances between graphs from two different models smaller than ϵ_k.

Precision (P) and recall (R) are then defined as :

$$P(\epsilon) = \frac{TP(\epsilon)}{TP(\epsilon)+FP(\epsilon)} \quad (2)$$

$$R(\epsilon) = \frac{TP(\epsilon)}{TP(\epsilon)+FN(\epsilon)} \quad (3)$$

The diagonals of $D_{1,1}$ and $D_{2,2}$ are trivial and are not considered (null distance between a graph and itself). To insure relevant computations of precision and recall, the diagonals of $D_{2,1}$ and $D_{1,2}$ are also removed. Given the symmetry of the GCD_{11}, $D_{1,1}$ and $D_{2,2}$ are also symmetrical and, $D_{1,2} = t(D_{2,1})$, where t means transpose. All counts are then twice larger than expected, which, however, simplifies when computing precision and recall. From the precision-recall curve, that is precision $P(\epsilon)$ as a function of recall $R(\epsilon)$, the AUPR is defined as:

$$AUPR = \sum_{k=2}^{100} P(\epsilon_k) \Delta R(\epsilon_k) \quad (4)$$

where $\Delta R(\epsilon_k)$ is the change in recall from rank $k-1$ to k. For each combination of order and edge density, the resultant AUPR is used to complete an $|N| \times |d|$ matrix of AUPR.

[illegible] AUPR score equal to 1 means a perfect distinction whereas an AUPR score equal to 0.5 represents a baseline which corresponds to the expected score of a random classifier. An AUPR score to 0 occurs when graph topologies are all identical. We arbitrary consider that an AUPR larger than 0.9 ensures a clear discrimination between two models. In the domain within which AUPR ≥ 0.9 further called domain of applicability, the GCD_{11}

is able to attribute small distance between graphs coming from the same model and large distance between graphs coming from different model. The complementary domain, called domain of uncertainty, corresponds to orders and edge densities for which the GCD_{11} lacks efficiency.

Comparing graphs with different order and edge density

In this second case, only ER and SF-BA comparisons are considered to test the ability of the GCD_{11} to assign smaller distances to pairs of graphs coming from the same models than to those coming from different models. We do not include REG in this approach because the topology of graphs coming from REG remains identical regardless of the order.

For all possible pairs of combinations of orders and densities $(N_1, d_1) \times (N_2, d_2)$ we build the three 100×100 following GCD_{11} matrices using the already simulated graphs:

$$\begin{aligned} D_{ER,ER}(N_1, d_1, N_2, d_2) &= (GCD_{11}(G^i_{ER}(N_1, d_1), G^j_{ER}(N_2, d_2)))_{i,j} \quad (5) \\ D_{SF\text{-}BA,SF\text{-}BA}(N_1, d_1, N_2, d_2) &= (GCD_{11}(G^i_{SF\text{-}BA}(N_1, d_1), G^j_{SF\text{-}BA}(N_2, d_2)))_{i,j} \quad (6) \\ D_{ER,SF\text{-}BA}(N_1, d_1, N_2, d_2) &= (GCD_{11}(G^i_{ER}(N_1, d_1), G^j_{SF\text{-}BA}(N_2, d_2)))_{i,j} \quad (7) \end{aligned}$$

We then compute the percentage of cases where the inter-model distance $D_{ER,SF\text{-}BA}(N_1, d_1, N_2, d_2)$ is larger than either of the two intra-model distances $D_{ER,ER}(N_1, d_1, N_2, d_2)$ and $D_{SF\text{-}BA,SF\text{-}BA}(N_1, d_1, N_2, d_2)$. This percentage is used to complete an $(N_1 \times d_1) \times (N_2 \times d_2)$ asymmetric matrix of probability. To limit computational and because the outputs change slowly with the order values, the numbers of possible values for the order are reduced so that $(N_1, N_2) \in \{5, 10, ..., 50\}^2$ and $(d_1, d_2) \in \{0, 0.01, ..., 1\}^2$. We arbitrary consider that a probability of at least 0.9 is sufficient to ensure a clear discrimination between two models which is the threshold used to defined the domain of applicability of the GCD_{11}.

Statistical test

In order to test if an empirical graph $G(N, d)$ is an outcome of an $ER(N, d)$ or an SF-$BA(N, d)$ random graph model the following randomized statistical test is built. First, we simulate independent outcomes M_k with $k = 1, ..., K = 1000$ of each possible reference model $M = ER(N, d)$ or SF-$BA(N, d)$ random graph model. Then, we compute their Graphlet Correlation Matrices $GCM(M_k)$ and their average:

$$\overline{GCM}_M = \frac{1}{K} \sum_{k=1}^{K} GCM(M_k) \quad (8)$$

We denote $\overline{GCM}_M$ the average Graphlet Correlation Matrix of M and buil[illegible]e test by computing η the number of times the distance between $GCM(G)$ and $\overline{GCM}_M$ is smaller or equal than the distance between $GCM(M_k)$ and $\overline{GCM}_M$. The p-value [46] is defined by $\hat{p} = (\eta + 1)/(K + 1)$. The larger the p-value is, the less evidence against H_0.

Empirical graphs

The developments proposed in this paper are illustrated on small graphs describing pairwise relationships (the edges) among a set of vessels (the nodes) identified in a previous work [37] based on joint-movement analysis [47]. Two particular and contrasting fleets (group of vessels sharing same technical characteristics) are considered among those studied in [37] with twenty graphs each. Based on pair trawling, Fleet 1 is characterised by strong pairwise collaborative relationships and leads to graphs that are

strictly k-regular [39]. Conversely, Fleet 2 is characterised by ephemeral relationships due to encounters at sea that are random or assumed to be so, and provides graphs with unknown topological properties and of unknown types.

Results and Discussion

Efficiency of GCD-11 on small graphs

Same orders and densities (ER, $SF-BA$ and REG)

When comparing graphs coming from Erdős-Rényi (ER) and Barbási-Albert scale free (SF-BA) models, the domain of applicability ($AUPR \geq 0.9$) of the GCD_{11} is parabolic with regards to the order and the density (Fig 1a. The range of edge densities allowing a clear discrimination depends on the order and increases with graphs order. For instance, for an order of 15 and 30, the domain of applicability respectively spans a range of edge densities from 0.25 to 0.4, and from 0.05 to 0.8. Furthermore, a perfect discrimination ($AUPR = 1$) is gradually reached for graphs with more than 30 nodes, more and more irrespective of the edge density. Overall, the domain of applicability exhibits an asymmetrical surface. For a given order, our results show that the discrimination between ER and SF-BA random graphs model is generally better for the lower half range of edge density.

Fig 1. Quality of clustering (AUPR) for three pairs of models. (a) Erdős-Rényi vs Barbási-Albert scale free, (b) Erdős-Rényi vs 1-regular and (c) Barbási-Albert scale free vs 1-regular. For each pair of models, and for each order (from 4 to 50) and edge density (from 0 to 1) combination, the quality of clustering between 100 graphs of the two models is assessed by the Area Under the Precision Recall curve (AUPR). A maximum value of 1 corresponds to perfect discrimination. Empirical graphs from fleet 1 (red squares) and from fleet 2 (blue triangles) are projected according their features (order and edge density).

A trivial part of the domain of uncertainty corresponds to combinations of order and edge density that lead to the same graph regardless of the graph models (isomorphic graphs [48]). For instance, densities of 0 and 1 result in empty or complete graphs respectively, and lead to null AUPR values (null distance between each pair of graph). The trivial part of the domain of uncertainty is indeed symmetrical (black crosses; Fig 1a).

The rest of the domain of uncertainty is rather asymmetric. For very small densities (left side), the number of edges is insufficient to enable the emergence of significant different topological components. For very high densities (right side), the two topologies gradually converge towards complete graphs. These two effects decrease as graph order increases and connect under a certain order threshold (approximately 12-14 nodes).

When comparing graphs originated from the 1-regular model and the Erdős-Rényi or Barbási-Albert scale free models (Fig 1b and Fig 1c), only even values of orders from 4 to 50 are consistent with the 1-regular property, and their densities are totally determined by their orders. A single AUPR is thus attributed to each order. In both cases, the AUPR increases as a function of the order, quickly reaching a perfect value (AUPR = 1) with orders equal to 16 and 10 for ER and SF-BA cases respectively. The GCD_{11} can therefore be used with confidence to discriminate an 1-regular from an ER or SF-BA random graphs for any order above 8 nodes (AUPR $\geq$ 0.9). The high minimum quality of clustering for all tested orders (at least 0.7) is explained by the invariant topology of 1-regular graphs (couples of disconnected nodes) which leads to null values in matrix

distance. These null distances provide an incompressible number of true positives in the computation of the AUPR score.

Different orders and densities (ER and $SF-BA$)

When dealing with different orders and densities, the domain of applicability of the GCD_{11} turns out to depend first on the order. For equal orders (Fig 2b, block diagrams on the first bisector), the surface of the domain of applicability increases from 0.015 to 0.19 when the order increases from 15 to 50. This means that the edge density difference allowing a clear discrimination between ER and SF-BA is larger for "large" graphs.

Fig 2. Probability to correctly distinguish Erdős-Rényi and Barbási-Albert scale free graphs for different order and/or edge density.
Each block (i, j) concerns the comparison of an ER of order N_i and a SF-BA of order N_j, with edge density d_k and d_l respectively ranging from 0 to 1. Dashed lines in each block highlight comparison when $d_k = d_l$.
(a) Probability that $D_{ER,SF\text{-}BA}(N_i, d_k, N_j, d_l) >$
$max(D_{ER,ER}(N_i, d_k, N_j, d_l), D_{SF\text{-}BA,SF\text{-}BA}(N_i, d_k, N_j, d_l))$.
(b) Proportion of cells with a probability $P \geq 0.9$ under or above the diagonal (cells covered by diagonals does not counted). Their mean quantifies the surface of the domain of applicability of the GCD_{11}.

Compared to the reference cases where the two graphs are of the same order (block diagrams in Fig 2b), an increase of the order of one of the two graphs leads systematically to larger domains of applicability when the increase concerns the ER graph. For instance, starting with the comparison between $ER(20, .)$ and SF-$BA(20, .)$ with a domain of applicability equal to 0.08, the domain of applicability expends from 0.09 to 0.12 when the order of the ER graph increases (in column), while it flattens around 0.09 when the increase of order concerns the SF_BA graph (in row).

The domain of applicability is also systematically asymmetric favouring situations where the edge density of the SF-BA graph is larger than the edge density of the ER graph it is compared to, whatever their respective orders (Fig 2a)). The asymmetry that exists on average is, however, dependent of the edge densities. As a matter of fact, when the orders increase, the domain of applicability acquires a "violin" shape. The violin's body represents the major part of the domain of applicability and concerns the lower half range of edge density. It is asymmetric with regards to the first bisector which means that the range of densities allowing to distinguish ER and SF-BA is larger when their edge densities are small, and when SF-BA graphs are denser than ER. The violin's head represents the domain of applicability, also asymmetric, for high or very high edges densities ($d \geq 0.7$). However the asymmetry is reversed, that is, when ER graphs are denser than SF-BA. The violin's neck is the finest part of the domain of applicability and appears as a transition between the two previous parts (the body and the head). In the violin's neck the GCD_{11} is able to distinguish ER and SF-BA with very similar edges densities.

Empirical graphs comparison

Empirical graphs features

Empirical graphs used in this study are characterised by small orders ranging from 10 to 25 nodes and large edge densities ranging from 0.05 to 0.61 (Table.1). Graphs of fleet 1 are on average smaller and strongly less dense than graphs of fleet 2. The two fleets from which the graph are built get substantial different graphs. On the one hand, due to a strong and exclusive collaborative relationship, fleet 1 (Fig 3a) leads to regular

graphs of degree 1, i.e, disconnected edges. On the other hand, graphs of fleet 2 (Fig 3c) show a single dense component reflecting multiple relationships. The peculiar 1-regular topology of graphs of fleet 1 results in a strong negative correlation between order and density which does not exist in fleet 2. As a matter of fact, 1-regular graphs gets even number of nodes and their sizes ($S = N/2$).

Table 1. Main features of empirical graphs: order (number of nodes), size (number of edges) and edge density (ratio between the size and the graph maximum size).

	Fleet 1			Fleet 2		
Graph	Order (N)	Size (S)	Density (d)	Order (N)	Size (S)	Density (d)
Graph_01	10	5	0.11	16	52	0.43
Graph_02	12	6	0.09	16	50	0.42
Graph_03	12	6	0.09	17	43	0.32
Graph_04	12	6	0.09	18	40	0.26
Graph_05	14	7	0.08	19	38	0.22
Graph_06	14	7	0.08	20	89	0.47
Graph_07	14	7	0.08	20	84	0.44
Graph_08	16	8	0.07	23	74	0.29
Graph_09	18	9	0.06	24	137	0.5
Graph_10	22	11	0.05	25	184	0.61
Mean	14.4	7.2	0.08	19.8	79.1	0.4
Range	[10 ; 22]	[5 ; 11]	[0.05 ; 0.11]	[16 ; 25]	[38 ; 184]	[0.22 ; 0.61]

Fig 3. Illustration of empirical graphs and their Graphlet Correlation Matrices. (a) Graph from fleet 1 and (c) from fleet 2. Nodes correspond to fishing vessels and edges to their relationships. The graph from fleet 1 contains disconnected edges reflecting exclusive pairwise relationships. The graph from fleet 2 contains a single dense component reflecting multiple relationships. (b) The Graphlet Correlation Matrix (GCM) of graph from fleet 1 and (d) from fleet 2. The 11 non redundant orbits are grouped according to their *role*, orbit $\{0\}$ represents the familiar degree, $\{2, 5, 7\}$ represent node in chain, $\{8, 10, 11\}$ represent node in cycle, and $\{6, 9, 4, 1\}$ represent terminal node. Cell colours correspond to the value of the correlation coefficient between the 11 non redundant orbits from 1 (yellow) to -1 (blue).

Due to the differences in degree and edge density, their respective GCMs also show major differences. The GCM of fleet 2 (Fig 3d) exhibits a standard shape [25] with strong positive and negative correlations between the first eleven non redundant orbits. These contrasted correlations capture heterogeneity in the role of vessels (nodes) in the graph. For instance, the negative correlation between orbits $\{4, 6, 9\}$ and orbits $\{0, 2, 5, 7, 8, 10, 11\}$ indicates the existence of peripheral nodes [25]. The GCM of fleet 1 (Fig 3b) shows a singular shape with a unit correlation between each pair of orbits. Indeed, in 1-regular graphs, and for all strongly k-regular graphs [49], each node has the same *role*, leading to the same eleven first orbits' degrees. This result suggests that regular graphs have the same GCM and consequently, cannot be distinguished using this metric.

Testing model type

All graphs of fleet 2 (blue triangles) (Fig 1b) are in the domain of applicability (AUPR ≥ 0.9). However, Graph 01, 02 and 10 are very close to the boundary of the domain of applicability of the GCD_{11}. The diagrams of AUPR presented in Fig 1b and Fig 1c

are specifically relevant for features of fleet 1 graphs that also lie in the domain of applicability of GCD_{11} (red squares). Consequently, it is relevant to use the GCD_{11} to test if empirical graphs are outcomes of ER of SF-BA random graph models.

None of the graphs from fleet 1 present any similarity with same order and density Erdős-Rényi or Barbási-Albert scale free graphs (Table 2). Due to the 1-regular topology of graphs from fleet 1, and according to their order from 10 to 22, theses results were easily predictable according to previous results on Fig 1b and 1c. Conversely, all graphs from fleet 2 are statistically not different from Erdős-Rényi graphs with an estimate p-value from 0.097 to 0.714. This suggests that graphs from fleet 2 and outcomes of Erdős-Rényi share similar topological properties. Edges, and by extension the relationships between vessels of fleet 2 , may be considered as statistically independent.

Table 2. Estimated p-values. Each empirical graph is associated to an estimated $p-value$ ($\hat{p}$) of being an outcome of an Erdős-Rényi or a Barbási-Albert scale free model. As in Table 1, empirical graphs are sorted according to their order. ($\hat{p}^{*} < 0.05$, $\hat{p}^{**} < 0.01$ and $\hat{p}^{***} \leq 0.001$)

	Erdős-Rényi		Barbási-Albert scale free	
Graph	Fleet 1	Fleet 2	Fleet 1	Fleet 2
Graph 01	0.002**	0.190	0.005**	0.001***
Graph 02	0.001***	0.571	0.001***	0.005**
Graph 03	0.001***	0.192	0.001***	0.001***
Graph 04	0.001***	0.714	0.002***	0.002**
Graph 05	0.001***	0.149	0.001***	0.014**
Graph 06	0.001***	0.107	0.001***	0.160
Graph 07	0.001***	0.097	0.001***	0.009**
Graph 08	0.001***	0.082	0.001***	0.001***
Graph 09	0.001***	0.293	0.001***	0.001***
Graph 10	0.001***	0.094	0.001***	0.572

However, Graphs 06 and 10 from fleet 2 also present a significant probability to be an outcome of Barbási-Albert scale free graphs ($\hat{p} \geq 0.16$). For Graph 06, the balanced p-value between ER ($\hat{p} = 0.107$) and SF-BA ($\hat{p} = 0.16$) may suggest that Graph 06 presents an intermediate topology between ER and SF-BA graphs. Indeed, the $AUPR$ ($1 > AUPR \geq 0.9$) associated to features of Graph 06 on Fig 1a implies small overlapping between ER and SF-BA graphs which does not exclude the existence of "extreme" graphs from these models which might present some similarities. Graph 06 might be one of these "extreme" graphs. For Graph 10, the unbalanced p-values between ER ($\hat{p} = 0.094$) and SF-BA ($\hat{p} = 0.572$) reflects a different situation. Even if the $AUPR$ associated to features of Graph 10 ($1 > AUPR \geq 0.9$) implies small overlapping between ER and SF-BA graphs, Graph 10 is also the most dense empirical graph ($d = 0.61$). According to this density, its small similarity with ER graphs could reflect the beginning of the topology convergence between the two models.

Pair testing

The objective here is to test if two empirical graphs are an outcome of the same random model or not. This could be helpful if the previous statistical test fails to identify significant similarities with any random graphs models. Based on previous results, we first identify the pairs of graphs that, given their respective orders and edge densities, belong to both sides of the domain of applicability of the GCD_{11}. This leads to consider the following four pairs of graphs: $\{(03;08);(04;05);(04,08);(05;08)\}$ (Fig 4). Not surprisingly, these graphs present small densities (from 0.22 to 0.32) and, in each of these pair, the two graph densities are very similar with a maximum density variation of

0.07 in pair (05; 08).

Fig 4. Probability to correctly distinguish Erdős-Rényi and Barbási-Albert scale free graphs with orders and edge densities of graphs from fleet 2. Each pair of empirical graphs (i, j) from fleet 2 is associated to a comparison of an ER of order N_i and edge density d_i and a $SF\text{-}BA$ of order N_j and edge density d_j. Each cell is colored as the probability that $D_{ER,SF\text{-}BA}(N_i, d_k, N_j, d_l) > max(D_{ER,ER}(N_i, d_k, N_j, d_l), D_{SF\text{-}BA,SF\text{-}BA}(N_i, d_k, N_j, d_l))$.

For each pair of graphs, the two intra-model distance distributions (ER vs ER) and ($SF\text{-}BA$ vs $SF\text{-}BA$) are very similar and overlap each other (Fig 5). This suggests that the GCD_{11} remains almost unchanged when comparing graphs coming from the same graph model for any graph model. On the other hand, the inter-model distance distribution (ER vs $SF\text{-}BA$) is clearly different and greater than the two intra-model distance distributions. However, there is a small overlap between these three distributions which is reflected in the probability values $1 > P \geq 0.9$.

Except for the pair (03; 08) (Fig 5a), the GCD_{11} between empirical graphs (red dotted lines) falls near the mode of the two intra-model distance distributions indicating that these graphs are likely to come from the same model. It is worth noting that, without the previous statistical test results (Table 2), this second test does not allow to identify if empirical graphs are an outcome of Erdős-Rényi or Barbási-Albert scale free graphs. However, this approach is relevant if the statistical test failed to identify significant similarities with any random graphs models by providing an alternative way to assess if two empirical graphs could be an outcome of the same model.

Fig 5. Distance between empirical graph from fleet 2. The dotted red line shows the distance GCD_{11} between each pair of empirical graphs from fleet 2 which presents suited features (order and edge density) to be compared. For each comparison, the empirical distance is compared with the two intra model distance distribution (ER vs ER in white, $SF\text{-}BA$ vs $SF\text{-}BA$ in black) and the inter model distance distribution (and ER vs $SF\text{-}BA$ in grey) computed according to features of pairs of empirical graphs.

Conclusion

This work extends the use of the graphlet correlation distance originally proposed for large real-world graphs to small real-world graphs. Through a numerical benchmark study, we show the relevance of the Graphlet Correlation Distance (GCD_{11}) for comparing graphs with the same order and the same density configuration. The generic statistical test proposed in this study to test the similarity between empirical graphs and graph models regardless of order and edge density can be applied without restriction on the size of the graphs. Some limitations of the GCD_{11} are highlighted on the basis of numerical evidences presented here. While the k-regular graphs defy any relevant comparison, the performance of the GCD_{11} deteriorates when the orders and/or the densities differ, especially with large density variations. This work is based on two contrasted and commonly encountered random graph models, the Erdős-Rényi and Barbási-Albert scale free graph models. However, the proposed experimental design and numerical analysis can be directly used with other random graph models to explore new properties of the GCD_{11} and extend its domain of applicability. For example, it might be interesting to explore the ability of the GCD_{11} to compare graphs with communities using the Lancichinetti-Fortunato-Radicchi [50] random graph model. The application of the

method developed in this study to fisheries data is particularly suitable for testing whether certain fishing behaviors can be considered independent. This property is generally required to apply statistical inference methods and more particularly when estimating population biomasses of marine ecosystems. A very operational goal of the GCD and the associated statistical test developed here could therefore be to identify the sub-part of the fishing data corresponding to this independence property and their use to provide an index of population abundance. Finally, by extending the use of GCD to small real-world graphs, we hope to stimulate research interest in graph-theoretic methods for these small graphs that are little studied in the literature.

Acknowledgements

We thank Sophie Lanco-Bertrand and Julien Lebranchu (IRD-Sète) for helpful comments and discussions. Authors would like to thank the SIH (Système d'informations Halieutiques-IFREMER) for the French fleet dataset. This work was supported by the French Region Pays de la Loire and the research project TRACFLO.

References

1. Scharf HR, Buderman FE. Animal movement models for multiple individuals. Wiley Interdisciplinary Reviews: Computational Statistics. 2020;12(6):e1506.

2. Hobson EA, Silk MJ, Fefferman NH, Larremore DB, Rombach P, Shai S, et al. A guide to choosing and implementing reference models for social network analysis. Biological Reviews. 2021;96(6):2716–2734.

3. Butts CT. Revisiting the Foundations of Network Analysis. Science. 2009;325:414–416.

4. Mukherjee C, Mukherjee G. Role of adjacency matrix in graph theory. IOSR Journal of Computer Engineering. 2014;16(2):58–63.

5. Pržulj N. Protein-protein interactions: Making sense of networks via graph-theoretic modeling. BioEssays. 2011;33:115–123.

6. Aspillaga E, Arlinghaus R, Martorell-Barceló M, Barcelo-Serra M, Alós J. High-Throughput Tracking of Social Networks in Marine Fish Populations. Frontiers in Marine Science. 2021;8:688010.

7. Zelinka B. On a certain distance between isomorphism classes of graphs. Časopis pro pěstováni matematiky. 1975;100(4):371–373.

8. Emmert-Streib F, Dehmer M, Shi Y. Fifty years of graph matching, network alignment and network comparison. Information sciences. 2016;346:180–197.

9. Wills P, Meyer FG. Metrics for graph comparison: A practitioner's guide. PLOS ONE. 2020;15:e0228728.

10. Soundarajan S, Eliassi-Rad T, Gallagher B. A Guide to Selecting a Network Similarity Method. In: Proceedings of the 2014 SIAM International Conference on Data Mining; 2014. p. 1037–1045.

11. Bounova G, de Weck O. Overview of metrics and their correlation patterns for multiple-metric topology analysis on heterogeneous graph ensembles. Physical Review E. 2012;85:016117.

12. Britton T, Deijfen M, Martin-Löf A. Generating simple random graphs with prescribed degree distribution. Journal of statistical physics. 2006;124(6):1377–1397.

13. Milo R, Shen-Orr S, Itzkovitz S, Kashtan N, Chklovskii D, Alon U. Network motifs: simple building blocks of complex networks. Science. 2002;298(5594):824–827.

14. Holland PW, Leinhardt S. Local structure in social networks. Sociological methodology. 1976;7:1–45.

15. Willett J. Similarity and clustering in chemical information systems. John Wiley & Sons, Inc.; 1987.

16. Van Wijk BC, Stam CJ, Daffertshofer A. Comparing brain networks of different size and connectivity density using graph theory. PloS one. 2010;5(10):e13701.

17. Faisal FE, Meng L, Crawford J, Milenković T. The post-genomic era of biological network alignment. EURASIP Journal on Bioinformatics and Systems Biology. 2015;2015(1):1–19.

18. Krause S, Mattner L, James R, Guttridge T, Corcoran MJ, Gruber SH, et al. Social network analysis and valid Markov chain Monte Carlo tests of null models. Behavioral Ecology and Sociobiology. 2009;63(7):1089–1096.

19. Croft DP, James R, Krause J. Exploring animal social networks. Princeton University Press; 2008.

20. Wey T, Blumstein DT, Shen W, Jordán F. Social network analysis of animal behaviour: a promising tool for the study of sociality. Animal behaviour. 2008;75(2):333–344.

21. Sih A, Spiegel O, Godfrey S, Leu S, Bull CM. Integrating social networks, animal personalities, movement ecology and parasites: a framework with examples from a lizard. Animal behaviour. 2018;136:195–205.

22. Croft DP, Madden JR, Franks DW, James R. Hypothesis testing in animal social networks. Trends in ecology & evolution. 2011;26(10):502–507.

23. Pinter-Wollman N, Hobson EA, Smith JE, Edelman AJ, Shizuka D, De Silva S, et al. The dynamics of animal social networks: analytical, conceptual, and theoretical advances. Behavioral Ecology. 2014;25(2):242–255.

24. Tantardini M, Ieva F, Tajoli L, Piccardi C. Comparing methods for comparing networks. Scientific Reports. 2019;9:17557.

25. Yaveroğlu ON, Malod-Dognin N, Davis D, Levnajic Z, Janjic V, Karapandza R, et al. Revealing the Hidden Language of Complex Networks. Scientific Reports. 2015;4:4547.

26. Pržulj N, Corneil DG, Jurisica I. Modeling interactome: scale-free or geometric? Bioinformatics. 2004;20(18):3508–3515.

27. Dimitrova T, Petrovski K, Kocarev L. Graphlets in Multiplex Networks. Scientific Reports. 2020;10:1928.

28. Milenković T, Filippis I, Lappe M, Pržulj N. Optimized null model for protein structure networks. PLoS One. 2009;4(6):e5967.

29. Ahmed NK. Graphlet decomposition: framework, algorithms, and applications. Knowledge and Information Systems. 2017;50:689–722.

30. Pržulj N. Biological network comparison using graphlet degree distribution. Bioinformatics. 2007;23:e177–e183.

31. Erdös P, Rényi A. On Random Graphs I. Publicationes Mathematicae Debrecen. 1959;6:290–297.

32. Gu J, Jost J, Liu S, Stadler PF. Spectral classes of regular, random, and empirical graphs. Linear algebra and its applications. 2016;489:30–49.

33. Newman MEJ. The Structure and Function of Complex Networks. SIAM Review. 2003;45:167–256.

34. He D, Jin D, Chen Z, Zhang W. Identification of hybrid node and link communities in complex networks. Scientific reports. 2015;5(1):1–14.

35. Hunter DR, Krivitsky PN, Schweinberger M. Computational statistical methods for social network models. Journal of Computational and Graphical Statistics. 2012;21(4):856–882.

36. Dunne JA, Williams RJ, Martinez ND. Food-web structure and network theory: the role of connectance and size. Proceedings of the National Academy of Sciences. 2002;99(20):12917–12922.

37. Joo R, Bez N, Etienne MP, Marin P, Goascoz N, Roux J, et al. Identifying partners at sea from joint movement metrics of pelagic pair trawlers. ICES Journal of Marine Science. 2021;78(5):1758–1768.

38. Barabási AL, Albert R, Jeong H. Mean-field theory for scale-free random networks. Physica A: Statistical Mechanics and its Applications. 1999;272(1-2):173–187.

39. Hubaut XL. Strongly regular graphs. Discrete Mathematics. 1975;13:357–381.

40. Davis J, Goadrich M. The relationship between Precision-Recall and ROC curves. In: Proceedings of the 23rd international conference on Machine learning - ICML '06; 2006. p. 233–240.

41. Spearman C. The Proof and Measurement of Association between Two Things. The American Journal of Psychology. 1987;100(3/4):441–471.

42. Barabási AL, Albert R. Emergence of Scaling in Random Networks. Science. 1999;286:509–512.

43. Van Mieghem P, Wang H, Ge X, Tang S, Kuipers FA. Influence of assortativity and degree-preserving rewiring on the spectra of networks. The European Physical Journal B. 2010;76:643–652.

44. Lozin VV, Milanič M. A polynomial algorithm to find an independent set of maximum weight in a fork-free graph. Journal of Discrete Algorithms. 2008;6:595–604.

45. Poncela J, Gómez-Gardeñes J, Floría LM, Sánchez A, Moreno Y. Complex Cooperative Networks from Evolutionary Preferential Attachment. PLoS ONE. 2008;3:e2449.

46. Davison AC, Hinkley DV. Bootstrap methods and their application. 1. Cambridge university press; 1997.

47. Joo R, Etienne MP, Bez N, Mahévas S. Metrics for describing dyadic movement: a review. Movement Ecology. 2018;6:26.

48. Gibbons A. Algorithmic graph theory. Cambridge university press; 1985.

49. Brouwer AE, Haemers WH. Strongly regular graphs. In: Spectra of Graphs. Springer; 2012. p. 115–149.

50. Lancichinetti A, Fortunato S, Radicchi F. Benchmark graphs for testing community detection algorithms. Physical review E. 2008;78(4):046110.

Figure_1

Click here to access/download;Figure;Fig1.tiff

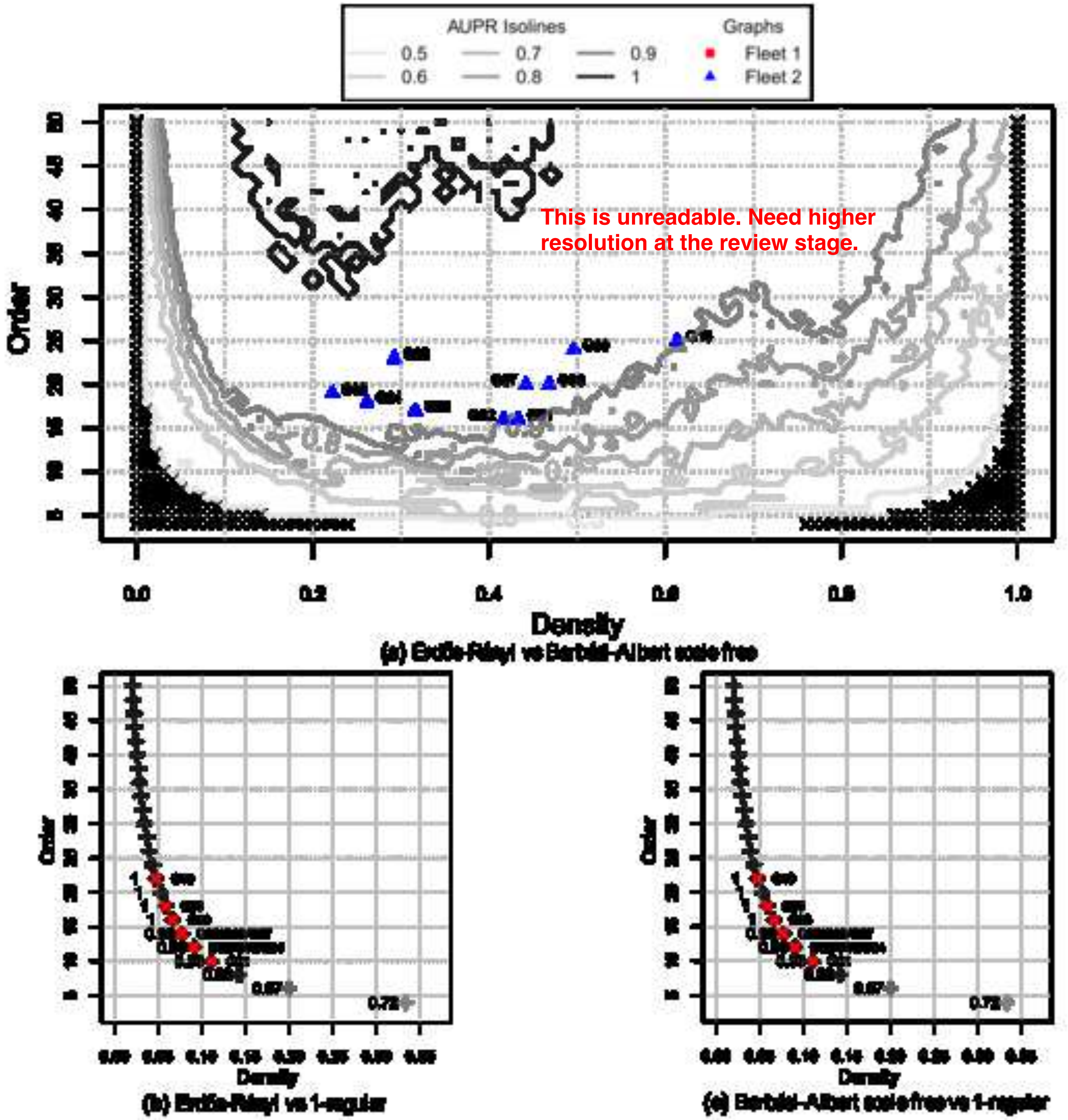

Is this more Figure 1 or is it
Figure 2? Labels!

Figure_2 Click here to access/download;Figure;Fig2.tiff

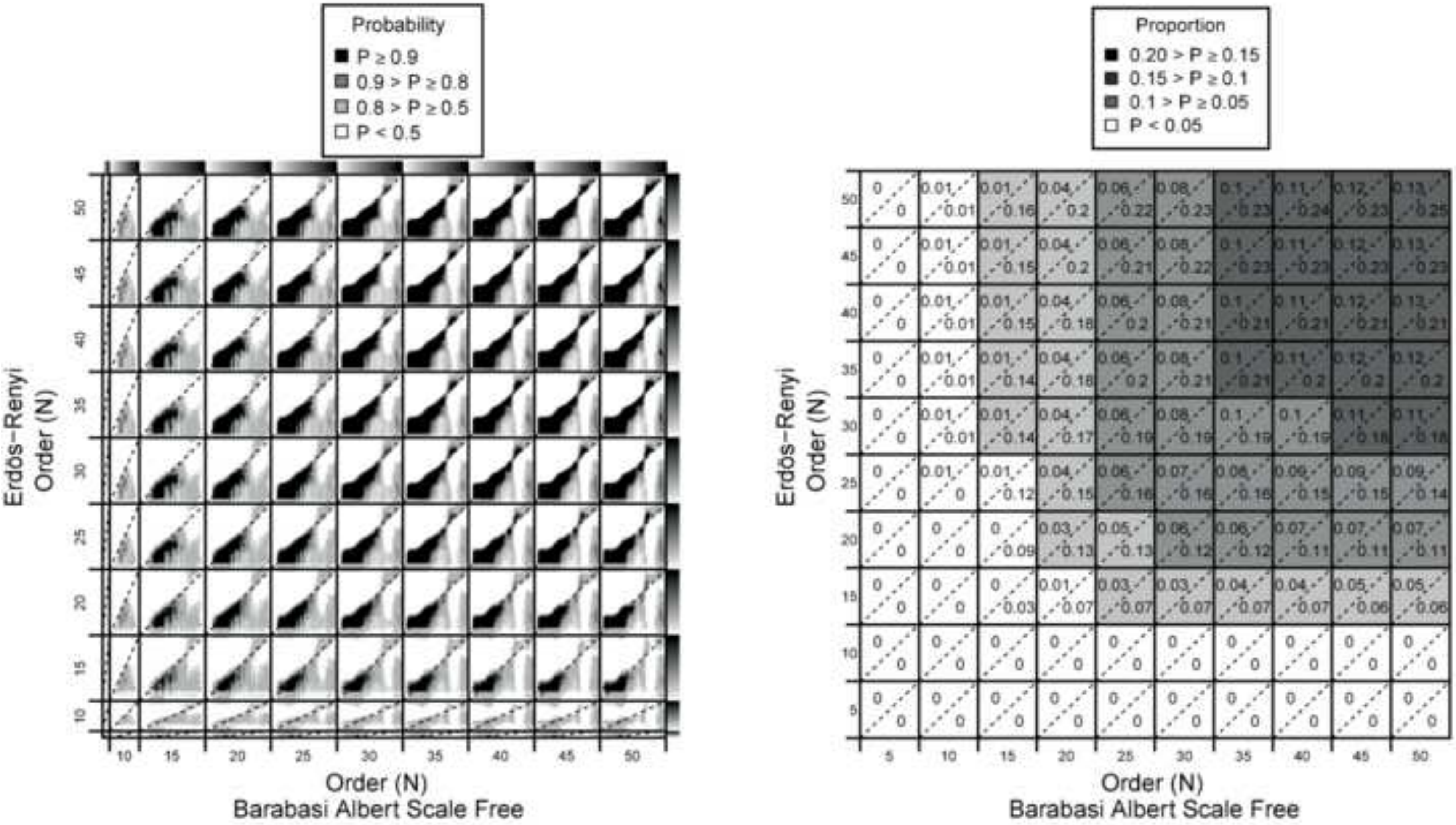

(a) Probability matrix

(b) Proportion matrix

Figure_3 Click here to access/download;Figure;Fig3.tiff

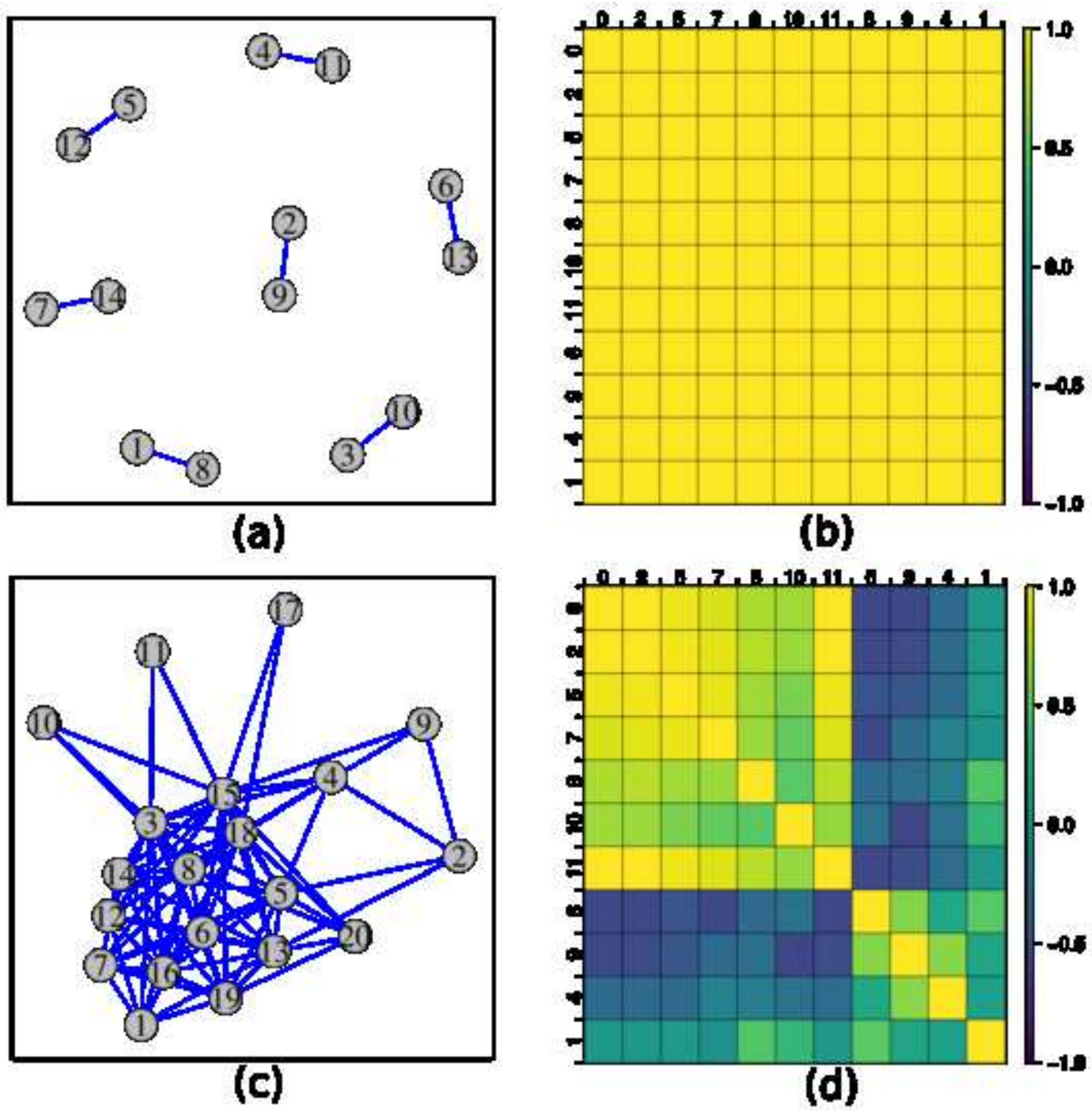

Figure_4

Click here to access/download;Figure;Fig4.tiff

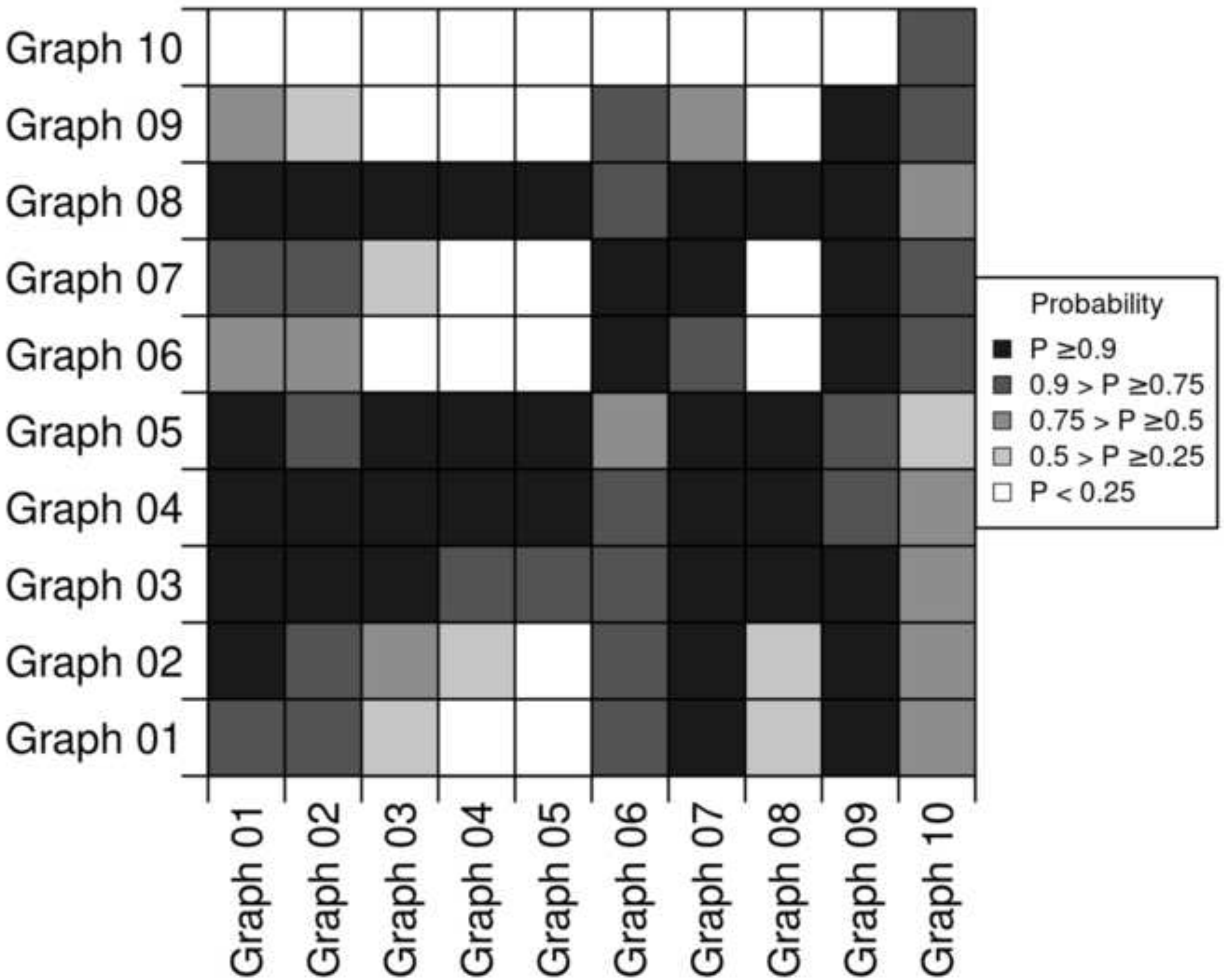

Figure_5

Click here to access/download;Figure;Fig5.tiff

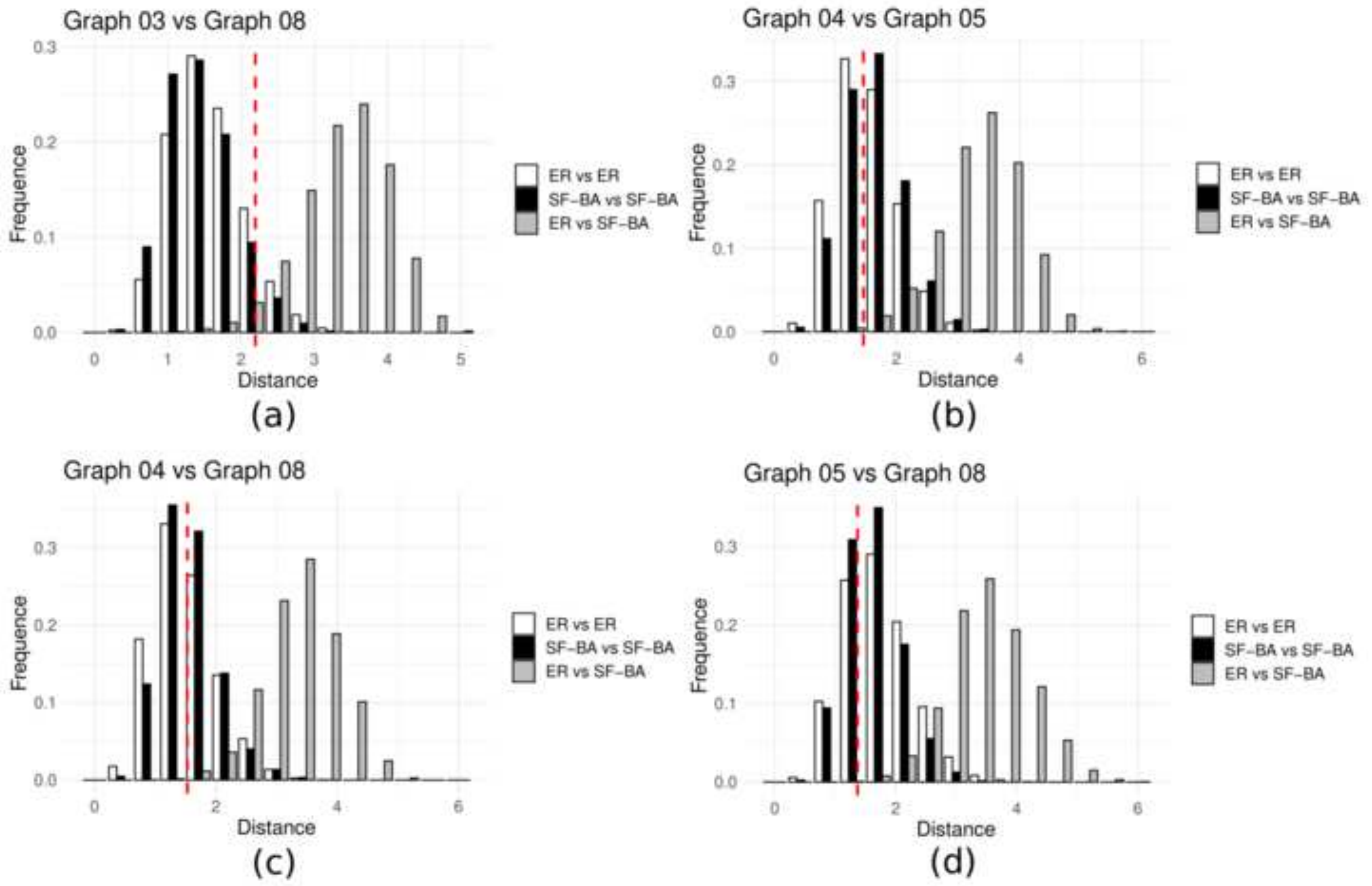

Supplement: Attachment — Submitted filename: GCD-on-small-graphs-PONE-D-22-11020_reviewer.pdf [file pone.0281646.s005.pdf]
