# Supplementary material for: Graphlet correlation distance to compare small graphs
Source: PLoS One. 2023 Feb 15;18(2):e0281646. doi: 10.1371/journal.pone.0281646 (PMC9931116; doi:10.1371/journal.pone.0281646)
Supplement: Attachment — Submitted filename: Response to Reviewers.pdf [file pone.0281646.s006.pdf]

# Response to Reviewers
# Graphlet correlation distance to compare small graphs

Jérôme Roux[1*], Nicolas Bez[2], Paul Rochet[3], Rocío Joo[4], Stéphanie Mahévas[1]

**1** UMR DECOD, IFREMER, BP 21105, 44311 Nantes Cedex 03, France
**2** MARBEC, IRD, Univ Montpellier, Ifremer, CNRS, INRAE, Sète, France
**3** ENAC, 7 av Edouaud Belin, Toulouse, France
**4** Global Fishing Watch, Washington, DC 20036, USA

* jerome.th.roux@hotmail.com

| Reviewer | Reviewer's comments | Author's Responses |
| --- | --- | --- |
| 1 | 1.1.<br>While the initial *inspiration* is brilliant, the implementation and (lack of) analysis feels like the result of a summer project from an undergrad. While the system is certainly a clever and novel, there's virtually no *insight* provided. They simply build empirical p-values, AUPRs, and a few simple (though reasonable) statistical tests, and then walk the reader through a bunch of observations of the resulting plots without providing any in-depth analysis or insight that can be gained. There's so much more low-hanging fruit they could explore without much effort—for example the analytical p-values I mention below... but they could also try to *explain* a bit about the GCM's and GCD's among ER graphs, using a few graphlets and orbits as examples. | 1.1.<br>We thank the reviewer for his suggestions and motivating comments.<br>We followed the reviewer's suggestion and went deeper in explaining the GCD distance: we explored the effect of the order and the density on the distribution of the 55 Graphlet correlation coefficients for both the Erdős-Rényi and Barbási-Albert scale-free models.<br>This additional analysis helped to understand the reasons for the success or failure of the GCD distance to discriminate the models in the same order and density configuration or in a different order and/or density configuration to some extent. (Lines 197 to 220 and Fig 2.). |
| | 1.2.<br>For Equation (8), my guess is each element (i,j) of the GCM's will be normally distributed with mean equal to the mean of your $\overline{GCM_M}(i,j)$. Why not compute the standard deviations as well, at which point you can compute analytical p-values for each element of a particular $\overline{GCM_M}(i,j)$ Then you can take the largest (or smallest) p-value and perform a Bonferroni correction (or use Empirical Brown's Method) to gather an *analytical* p-value? Then compare the analytical one with the one you use here and explain any differences. | 1.2.<br>We used a Shapiro-Wilk test to test the normality of each element (i,j) of the GCM's on a set of 100 or 1 000 Erdős-Rényi or Barbási-Albert scale-free graphs and for different values of order and density. Even if for a given order and a given density, some elements (i,j) respond positively to the normality test, it is not always the case, and there is no clear pattern of normality associated with densities or models. In other words, the evidence does not favour a normality assumption that would allow using the procedure suggested by the reviewer to obtain an analytical p-value.<br>On the other hand, the idea of considering the standard deviation of each element (i,j) is relevant. That's why, we improved our statistical test by computing the standardised distance GCD11, standardised by the standard deviation of each element (i,j). (Lines 320 to 330, equation SI.1, and Table SI.1.). |
| | 1.3.<br>I think 0.5 only represents random when there are two classes; you have three. If there are N classes I think 1/N is the expected AUPR of a random classifier. | 1.3.<br>Yes, we have 2 intra-model distances and 1 inter-model distance. However, for AUPR, we only have 2 qualitative classes: "Distance between graphs from the same graph model" and "Distance between graphs from different graph models". For these two classes, it is OK to consider 0.5 as random. |

| | | |
|---|---|---|
| | 1.4.<br>Finally.... PLEASE include better figures in the next round. The resolution is TERRIBLE and the figures are virtually illegible... and not numbered! And please put the figures IN LINE, ABOVE THE CAPTIONS. Putting figures at the end is a 20th-century practice and may be required by the journal once the paper is ACCEPTED... but at the review stage it's a bloody pain in the ass to flip back-and-forth from the back of the paper to the figure caption. | 1.4<br>We are sincerely confused about this inconvenience. The resolution of all the figures has been increased.<br>All the figures are now in line and above the captions. |
| 2 | 2.1.<br>The authors correctly discuss the fact that for low and high edge densities, especially for small graph orders, ER and BA-SF models cannot be distinguished by GCD nor probably by any other means since such graphs are essentially the same. Perhaps this line could be emphasized somewhat more in the discussion. Is there any theoretical literature covering the question of "when" (in terms of edge density) the graph topology of small graphs becomes truly distinct from random graphs? | 2.1<br>There are very few references on small graphs and, to our knowledge, there are no references on joint transitions between 2 models. There are only references concerning phase transitions within a model, such as the paper "on the evolution of random graphs" by P.Erdős and A.Rényi, cited in the manuscript. |
| | 2.2.<br>I also think that the authors should have investigated in depth one example where GCD fails to reliably distinguish between ER or BA-SF generated instances of different edge densities. This could be visualized by embedding GCMs in 2D using say UMAP or tSNE and then further considered by looking at a few particular GCMs and orbits. This would provide a deeper understanding the limitations and strengths of GCD. | 2.2.<br>See response 1.1. for a first element of answer. We have also added a series of PCAs to visualise the effect of increasing the difference in graphs' densities. We considered that PCA was more relevant than UMAP or tSNE for this visualisation step. On the one side, because PCA is based on an Euclidean distance like the $GCD$, and on the other side, because the distances between graphs are preserved in the projection. (Lines 237 to 247 and FIG 4.). |
| | 2.3.<br>Finally, the authors may want to include the following paper as an example of an application of GCD. A method called correlation graphlet analysis (CGA) is proposed there (details in supplement, code available at https://github.com/astojmir/cgamodules) to select robust correlation thresholds in gene co-expression networks and derive modules from them. | 2.3.<br>Sorry but we have not found any reference to the paper mentioned, not even through the git repository. |
| | 2.4.<br>There is also a typo or omission on the lines 169 and 170 - "computational ..." - a word is possibly missing. | 2.4.<br>Yes. Done (Line 152). |

| | | |
|---|---|---|
| 3 | 3.1.<br>To measure the significance of the topological fit of a given network to a model network, the authors design a test to compute an empirical p-value (i.e., how likely is it that I measure a GCD distance of 'x', between the network and generated model networks by chance). Although methodologically correct, computing an empirical p-value is quite trivial and hence the methodological contribution of this paper is very minor. All the more as a Mann-Whitney-U test has already been designed and applied for the same thing (see [1], Windels, S. F., Malod-Dognin, N., & Pržulj, N. (2019). Graphlet Laplacians for topology-function and topology-disease relationships. Bioinformatics, 35(24), 5226-5234). | 3.1.<br>Thank you for this very interesting reference. According to our understanding, the Mann-Whitney-U test allows to test under the null hypothesis H0, if two distributions are identical. However, in our statistical test we only have one distribution (the distribution of the distance $d_{M_k}$) and a single value $d_G$. For that reason, the Mann-Whitney-U test would not be suited for our purposes. |
| | 3.2.<br>The presented analysis is incomplete. Firstly, although GCD is the state-of-the-art distance measure for large networks, it has not been shown to be so for small graphs (5-100 nodes), so a comparison w.r.t. other methods should be included. | 3.2.<br>We would like to clarify the position of this paper. The aim of this paper is not to identify the best method to compare small graphs. It is rather to know if the $GCD$ is able to compare small graphs, and if so, under what circumstances. Thus, considering that the GCD is the state-of-the art distance for large graphs, we were interested, at first for real practical problems, by its behaviour in the world of small graphs. By the way, it does not seem trivial to ask when a graph is a large graph with respect to the GCD. |
| | 3.3.<br>All the more so, as the statistical power of GCD for such small graphs is questionable. This, as GCD is based on correlations between graphlet counts, and computing the correlations over fewer than 20 data points (nodes) is unlikely to capture much signal. | 3.3.<br>We fully agree. This is precisely the main question of this paper: for which order and density ranges, is the GCD able to discriminate graphs from different models? Indeed, this paper indicates in which cases it does.<br><br>In other words, we are not questioning the effectiveness of the GCD, we are trying to define its domain of relevance for small graphs. |

| | | |
|---|---|---|
| | 3.4.<br>Lastly, the authors only consider three types of model networks in their analysis, which is very few considering the plethora of model networks available. For instance, the paper introducing GCD considered seven model networks. | 3.4<br>We agree. There is potentially a plethora of possible models. An exhaustive analysis is thus difficult to achieve.<br><br>We have chosen to limit ourselves to three models deemed relevant to key situations in eco-ethology. Erdős-Rényi models for graphs are analogous to Gaussian models for distributions, or to the Poisson process for point processes. In point processes, the Poisson point process is used as a baseline model of independence between points in space, regardless of the possibility to observe clusters of points. In graphs, the ER model can be used as a baseline model of edge independence; the goal is to test how inconsistent the existence of connections in a graph is with regard to the possibility that these connections appear at random and independently. The Barbási-Albert scale-free model was selected for its well-known properties and recurrent use in the literature. Finally, the $k$-regular model is relevant for graphs like those from fleet 1 where paired behaviours exist.<br><br>We consider that our results are not exclusive to these models. Indeed, due to our results on the behaviour of the Graphlet correlation coefficients (Fig.2.), we have good reasons to believe that our results on the domain of applicability (left and right side effects or minimum order threshold) can be, at least qualitatively, transposed to other pair of models (see lines 197 to 220).<br><br>Finally, making all our work reproducible was one of our priorities. In this way, all the codes are open access for anyone to use on the models of their choice. |

| | | |
|---|---|---|
| | 3.5.<br>The quality of writing in this paper is not up to a scientific standard, as many concepts are either vaguely defined, not defined or defined after they are first used. For instance, graphlets in this paper are defined as 'small connected sub-graphs that extend the concept of motifs'. Not only is this definition of graphlets wrong, graphlets are 'small connected non-isomorphic and induced subgraphs' [2], motifs have also not been defined in this paper, so the comparison is uninformative. Also, without an intuitive explanation, a non-expert reader will not understand what a graphlet is from this formal definition. | 3.5.<br>Concerning the definition of Graphlet, it is indeed a mistake that has been corrected; thanks for bringing it up. We added it in lines 28 to 30<br><br>We thank the reviewer for this comment. We have edited the manuscript accordingly and double checked that all definitions are provided when they are first used. We hope that with the addition of this key definition (i.e topology (line 16), motifs (line 18), order and density (line 26-27)...) and other edits in the revised version of the manuscript, we have improved readability. On the other hand, We have tried to produce a paper accessible to a broad scientific community (life sciences, environmental sciences, social sciences...). Consequently, we chose to avoid technical language that could be off-putting for non-experts in graph theory. However, we consider that we have provided enough references to allow the reader to deepen all the technical points discussed in this paper. |
