# Supplementary material for: Graphlet correlation distance to compare small graphs
Source: PLoS One. 2023 Feb 15;18(2):e0281646. doi: 10.1371/journal.pone.0281646 (PMC9931116; doi:10.1371/journal.pone.0281646)
Supplement: Attachment — Submitted filename: Response to Reviewers.pdf [file pone.0281646.s007.pdf]

Response to Reviewers
Graphlet correlation distance to compare small graphs

Jérôme Roux[1*], Nicolas Bez[2], Paul Rochet[3], Rocío Joo[4], Stéphanie Mahévas[1]

1 UMR DECOD, IFREMER, BP 21105, 44311 Nantes Cedex 03, France
2 MARBEC, IRD, Univ Montpellier, Ifremer, CNRS, INRAE, Sète, France
3 ENAC, 7 av Edouaud Belin, Toulouse, France
4 Global Fishing Watch, Washington, DC 20036, USA

* jerome.th.roux@hotmail.com

Reviewer	Reviewer's comments	Author's Responses
1	No comments	
2	2.1.<br>In this revision, the authors added an investigation about the variability of individual Graphlet correlation coefficients with respect to change in density and order. This view could potentially form a starting point for designing an improved distance measure between graphs, perhaps by incorporating terms related to degree or density.	2.1.<br>Thank you for your comment. A sentence has been added at the end of the MS opening this research avenue. (Lines 381-383)
	2.2.<br>This version is definitely an improvement to the previous version, addressing many of the reviewers' comments, but could probably go a bit further to understand the issues associated with using GCD (or any other graph topology similarity measure) for smaller graphs.	2.2.<br>Thank you for your comment. The current revision of the paper includes two new random graph models (Geometric and Watts-Strogatz small-world) widely used in graphs literature, on which we investigated the ability of the GCD_{11} to distinguish between graphs coming from different random graph models in the context of small graphs. We also change the name of the domain of relevance for using the GCD_{11}, in the density-order space (now the domain of separability) to be more accurate on the scope of this work. We certainly have shared more insights about on the use of the GCD_{11} for small graphs in the new revision.

3	3.1.<br>• The main conclusion of this paper is that 2 fishing fleets are of ER topology.<br>• To me, this conclusion could have been achieved by looking at the degree distributions and fitting them with a poisson distribution.	3.1<br>• We thank the reviewer for his comment. The paper focuses on a simulation-based study of the GCD_{11} and the case study comes as a second objective to illustrate the scope of the advances in the understanding of GCD_{11}. Therefore, the main conclusions of this paper concern the behaviour of the GCD_{11} on small graphs and the elicitation of the domain order x density which allows clear discrimination between models, and the development of a statistical test to assess the probability that a given graph could be an outcome of a given model. In the conclusion, we put now more emphasis on the simulation-based study of the GCD_{11}. We also added more explanations in the abstract and the introduction to clarify the focus of the paper (Lines 62 - 78).<br>• The reviewer is right in saying that we could use a test comparing the distribution of degrees to a Poisson distribution to test if the graph is ER, but only when the number of vertices is large. Indeed the distribution of degrees has a binomial distribution of parameters (N,p), N being the number of vertices. However, in this study, we are interested in small-size graphs and the purpose of this study is not to test if a graph is ER but to illustrate the use of the GCD_{11} to test if graphs are outcomes of a larger number of random graph models. For that reason, we do not use a Poisson distribution for testing.
	3.2.<br>The 'domain of applicability', defines combinations of graph sizes (in terms of number of nodes) and network densities where GCD can be used to distinguish networks of different topologies. This domain is determined for graphs with known and very specific topologies (ER graphs, SF graphs and k-regular graphs). I wonder how valid this boundary of applicability is for the comparison to the real world networks (fishing fleet networks), which are of unknown topology. In my opinion, the "boundary of applicability" is only applicable in a generic fashion if the authors were to consider a more varied range of model networks.	3.2.<br>We agree with the reviewer. As suggested by the reviewer, we have thus extended the analysis by adding other random graph models. In this revised version, the performance of the GCD_{11} is now assessed through 4 random graph models :<br>- The Erdős-Rényi<br>- Fitness Scale-Free (which replaces the Barbási-Albert model)<br>- Watts-Strogatz small-world<br>- The geometric model<br>We also changed the name of the domain (from domain of applicability to domain of separability) to be more accurate in the intended meaning of the domain which indeed is not as general as the previous name might have suggested.<br><br>We believe that these additions allow for defining a more robust and general domain of separability.

3.3.<br>From Figure 5, fleet 1 has a clear 1-regular structure. As no other orbit but orbit 0 (an edge) is ever touched, I would argue that the GCM is undetermined (a correlation between different orbit counts can not be determined if all orbit counts are simply 0). So, to me, Figure 5B is wrong, as the GCM for such a network is not an 11 by 11 matrix of 1's but one of NAN's. Secondly [...] . Again, an argument for why GCD simply is not applicable. Also, a network can not be of 1-regular topology and of ER topology, so the text is contradictory.	3.3.<br>The 1-regular graph is indeed very peculiar. The data corresponded to a key fleet to validate the process of building graphs of interactions between vessels from individual GPS tracking data because of its known paired collaborations. We used that fleet as a reference fleet in the first version of the paper. However, the GCM is undetermined as said by the reviewer. Since the purpose of the paper is not to investigate the type of interactions between the vessels and the k-regular graphs cannot be investigated with the GCD_{11}, we decided to remove this fleet from the analysis.
3.4.<br>As is clear from my previous comments, I don't think GCD is the correct tool here. It would help if the authors showed it actually performed better than other distance metrics (like simply the distance between degree distributions).	3.4.<br>We have probably not been clear enough in explaining the framework of our study. As pointed out in the response to the previous comment, the aim of this paper is not to identify the best method to compare small graphs. It is rather to know if the GCD_{11} is able to compare small graphs, and if so, under what circumstances. Thus, considering that the GCD_{11} is the state-of-the-art distance for large graphs, we were interested in its behaviour in the world of small graphs. And in particular in the context of graphs comparison with different order and density where the GCD_{11} is not very effective. We tried to make this clearer in the Introduction of the manuscript (Lines 62-78).
3.5.<br>The shift to quantitative graph comparisons with the introduction of similarity or distance measures is more recent[23]".<br>The paper referenced dates from 2014. Network distance measures have been introduced long before that. Even graphlet based network comparison was introduced before in 2004.	3.5.<br>This has been rephrased and we removed the temporal reference (Line 27-29).

<table>
<tr><td></td><td>3.6.
This comparison is often done in a descriptive and qualitative way by comparing synthetic indicators of graph topology i.e the configuration by which the individuals of a graph are connected.
→ i.e. is written two dots.
In fact, in many graphs, the node degree distribution seems to follow a power law whose power γ is comprised between 2 and 3.
→ Comprised is not the right word here.
On one hand, the increase in order allows the emergence of complex topologies consistent with the topological properties of the model ([52]); on the other hand, the Spearman's Correlation coefficient becomes more accurate when computed on a larger number of nodes ([53]); . . .
→ Why the brackets around the references?</td><td>3.6.
Thanks for these comments. We modified the text to address each one as follows :
Done (line 17)
Replaced by “between” (line 106)
It's a mistake, the brackets have been removed (line 245-247)</td></tr>
<tr><td></td><td>3.7.
Graphlets are small connected non-isomorphic (different number of nodes and/or connected in a different way [28])
→ I don't think anyone understands what non-isomorphic means in this context from this definition. Two nodes are isomorphic if, when you swap their node label (position in the network), you still end up with the same network/graphlet.
The authors mention that graphlets are an extension to motifs. How so? Graphlets are induced, motifs are not. The way it is written it just looks as if the authors don't know.</td><td>3.7.
We agree with the reviewer. We changed the definition to: Graphlets are small connected induced subgraphs defined up to isomorphism (meaning that two isomorphic induced subgraphs are considered as two occurrences of the same graphlet) (Line 32-36)
We removed this ambiguous part of the sentence (Line 32-36).</td></tr>
</table>

4	4.1.<br>However, the paper falls short of this goal in many ways. The "domain of applicability" with respect to number of nodes and graph density is established based on separability of Erdős-Rényi (ER) and Barabási-Albert (BA) random graph models, which is a very narrow application. Furthermore, small BA graphs make little sense; their key feature of power-law degree distribution is hardly apparent with graphs of 5-50 nodes, and the effect of the initial network on the result is non-negligible (what initial graph is used is not explained). Thus, the comparison of BA to ER doesn't make sense to begin with, and even if it did, their separability is not indicative of any general "domain of applicability" that could be used to inform whether GCD is applicable to any other separation or clustering task of arbitrary small graphs.	4.1.<br>We agree with the reviewer. To extend the scope of the domain and to break out of the narrowness of the comparison between the ER and the BA, first replaced the BA model with the Fitness scale-free model which does not include a growth process from an initial graph. Second, we extended the analysis by adding other random graph models: Fitness Scale-Free (which replaces the Barbási-Albert model), Watts-Strogatz small-world, and geometric model.<br><br>Also, in this revised version, we explored a wider range of order (from 10 to 100) which should allow the emergence of the power-law degree distribution. However, our results suggest that the differences in topologies appear very quickly (i.e. for small orders) allowing us to distinguish this model from the other three.<br><br>We also changed the name of the domain (from domain of applicability to domain of separability) to be more accurate in the intended meaning of the domain which indeed is not as general as the previous name might have suggested. Nevertheless, we agree that the domain of separability cannot "be used to inform whether GCD_{11} is applicable to any other separation or clustering task of arbitrary small graphs". However, it allows a discussion of the interpretation of the results of the statistical test. Finally, we believe that this is a good starting point for future studies: performing this kind of study over a larger number of models and identifying the domain of separability for them will contribute to improved knowledge of the usefulness of the GCD_{11} for separation or clustering of small graphs. We added a sentence in the Conclusion to encourage further studies in this direction (Line 379-383).
	4.2.<br>The paper proposes a statistical test to tell if a graph is similar to the ER or BA model. This is a simple simulated sampling p-value calculation based on simulations of the models, using GCD as the test statistic. Using GCD for this task makes little sense, especially for comparison with the ER model, where edges are independent. Since the "calculate p-value through simulations" process is elementary textbook material and the simulation results here are restricted to ER and BA models, there is no wider applicability to the statistical testing section.	4.2.<br>We respectfully disagree with the reviewer.<br>Firstly, our statistical test is a generic test, applicable to whatever the choice of the reference graph model and is not restricted to ER and BA. For instance, in this revised version, we use it on the Watts-Strogatz and Geometric models.<br>Secondly, we also propose a standardized version of the statistical test that takes into account the variability of orbits' values and opens the possibility of more sophisticated statistical tests.

4.3.<br>The empirical graphs section details two classes of fishing vessel graphs, the first of which consists of 1-regular graphs. For a 1-regular graph, the first graphlet degree (i.e. node degree) is 1 and every other graphlet degree is 0 for every node. It does not make any sense to use any graphlet-based methods on these graphlet degree distributions. All the graphs have identical highly degenerate graphlet degree counts. It makes absolutely zero sense to use GCD with these graphs. Earlier in the paper, 1-regular graphs are compared to simulated ER and BA with GCD as well, which is also nonsensical. Including nonsense results is unnecessary and they should be omitted.	4.3.<br>We agree with this comment which is similar to the comment of another reviewer. Fleet 1 has been removed in the revised version.
4.4.<br>With the second class of empirical fishing vessels, it is concluded that they are in the "domain of applicability". What is meant is that they are in the domain where ER and BA models can somewhat be separated from each other. This does not translate to applicability to any arbitrary graphs, such as these fishing vessel graphs.	4.4.<br>We agree that the domain of separability does not translate to the applicability to any arbitrary graphs. However, we now extended its use to four different types of models.<br>In addition, according to our statistical test, there are two possibilities:<br>- 1. Reject the null hypothesis: in this case, there is nothing more to do or say.<br>- 2. Support the null hypothesis: in this case, we think that taking into account the domain of separability provides more information to interpret and discuss the result.
4.5.<br>Throughout the paper, GCD-11 is used. This measure takes into account 11 specific orbits, but is not the only GCD measure possible. Any set of graphlets and their orbits can be used to construct a GCD. The analysis is therefore very limited. It would be better to look more at individual graphlet degrees and different subsets of graphlets chosen to be included in the GCD, and how all the different graphlet degrees vary in small graphs. More graph structures should be considered, not only ER and BA.	4.5.<br>It is not the purpose of this paper to test whether other GCD measures or other distance measures perform better. However, the detailed inspection of the behaviour of the 11 different orbits through the analysis of the GCM in a small-graphs context is a relevant contribution of our work (Line 234 - 251).

	4.6.<br>In the Conclusion section, it is stated that "However, due to the behaviour of the Graphlet [sic] correlation coefficients in response to change in density, we expect the domain of applicability between the Erdős-Rényi and Barbási-Albert [sic] scale-free graph models described in this work to be qualitatively generic to other pair [sic] of models allowing a density ranging from 0 to 1.". There is nothing in the paper to back up this claim and I would be highly surprised if it was true. Setting up a general domain of applicability for GCD in small graphs is the goal of this paper and the main claim regarding that is unsubstantiated.	4.6.<br>Again we agree with the reviewer that the domain of separability of the GCD_{11} established in this paper is dependent on the random graph models included in the analysis. To increase the generality of this domain of separability, we added 3 new random graph models to our analysis. To be more accurate we also specified that we do not pretend to describe the domain of separability of the GCD but the one of the GCD_{11} associated with the 4 simulated random graph models. However, the analysis of 6 domains of separability showed strong similarities between them, suggesting that this could be extended to other models or at least encouraging extending the investigation to other models (Line 224-228). The sentence has been rephrased as follows: *According to these four models we show that the behaviours of the graphlets correlation coefficient in response to changes in density remains qualitatively similar.* (Line 224-228)
