# Supplementary material for: Graphlet correlation distance to compare small graphs
Source: PLoS One. 2023 Feb 15;18(2):e0281646. doi: 10.1371/journal.pone.0281646 (PMC9931116; doi:10.1371/journal.pone.0281646)
Supplement: Attachment — Submitted filename: Response to Reviewers.pdf [file pone.0281646.s008.pdf]

Response to Reviewers
Graphlet correlation distance to compare small graphs

Jérôme Roux[1*], Nicolas Bez[2], Paul Rochet[3], Rocío Joo[4], Stéphanie Mahévas[1]

1 UMR DECOD, IFREMER, BP 21105, 44311 Nantes Cedex 03, France
2 MARBEC, IRD, Univ Montpellier, Ifremer, CNRS, INRAE, Sète, France
3 ENAC, 7 av Edouaud Belin, Toulouse, France
4 Global Fishing Watch, Washington, DC 20036, USA

* jerome.th.roux@hotmail.com

Reviewer	Reviewer's comments	Author's Responses
1	No comments	
2	No comments	
3	No comments	
4	4.1<br>Revision 2 is much, much better than revision 1. The analysis has been extended to more network models, and the text has been revised to more accurately reflect the content of the work. Revision 2 is in a state that it fulfills, to my knowledge, the criteria for publication in PLOS ONE.	4.1.<br>We sincerely thank the reviewer for noticing the improvement in our work!
	4.2.<br>Line 157-158: diagonals of $D_M 2, M1$ and $D_M 1, M2$ are removed, but I don't see why. The first element of the diagonal of $D_M 1, M2$ is the distance between model 1 graph 1 and model 2 graph 1, which are different graphs, and I don't see why their distance should be excluded. I don't understand the reason that is given ("Ensuring relevant computations"), so it would be good to give a valid reason as to why the diagonals have been removed.	4.2.<br>By removing the diagonals $D_{M1,M1}$ and $D_{M2,M2}$ (null distance between a graph and itself) we unbalance the number of intra-model distances ($D_{M1,M1}$ and $D_{M2,M2}$) according to the number of inter-model distances ($D_{M1,M2}$ and $D_{M2,M1}$) in the matrix D. We have therefore removed the diagonals $D_{M1,M2}$ and $D_{M2,M1}$ to get the same number of intra and inter-model distances in the matrix D. This is what we meant by "ensuring relevant computations", but this may not be necessary if the $AUPR$ is not sensitive to the difference in the number of intra or inter model distances... We have added these considerations to the text (Lines 158 to 162)
	4.3.<br>When talking about the order of a graph for the first time, it would be good to define it as the number of nodes, since "number of nodes" is more common terminology than "order" and some readers are probably unfamiliar with the latter (the first mention is in the abstract).	4.3.<br>We have chosen to use the terminology "order" because "number of nodes" would have made the text more cumbersome. We now specify in the abstract that order means "number of nodes" and keep that same information in the first use of "order" in the Introduction (Line 30).

	4.4.<br>From line 138 onwards: the sentence "For a given order N in $\{10, 11, ..., 50, 55, ..., 100\}$ with cardN = $\|N\| = 51$, and a given edge density d in 0,0.01,...,1 with cardd = $\|d\| = 101$ ..." has some issues. First, the range of N should be more clearly presented, such that the steps between consecutive values and the transition point are unambiguously clear (for example "N in $\{10, 11, ..., 48, 49, 50, 55, 60, 65, ..., 100\}$"). Secondly, N is the number of nodes (an integer, a member of the set 10,11,... and so on), so notation cardN isn't clear (a single integer is not a set) and notation $\|N\|$ also seems to refer to the absolute value of the number of nodes. What is actually meant seems to be that there were 51 different values for N (i.e. the size of 10,11,...,48,49,50,55,60,65,...,100). This should be just written out instead of using tricky notation (incorrectly).	4.4.<br>Thank you for this proposal and this example of reformulation. We simplified and rewrote this part in an unambiguous way : *We define a set of 51 possible values of order N describing two gradual incremental steps as* $N \in \{10, 11, ..., 48, 49, 50, 55, 60, ...90, 95, 100\}$ *and a set of 101 possible values of edge density d regularly spaced as* $d \in \{0, 0.01, ..., 0.99, 1\}$ (Line 139 to 142)
	4.5.<br>Equations 11 and 12: these are equations for graphlet correlation distance (GCD) between the meanGCM_M and $GCM_M k$ or GCM_G. It would be helpful to write that in the text, so the reader is aware that this equation actually computes GCD	4.5.<br>We have specified that it is indeed the GCD (Line 205 to 207)
	4.6.<br>Line 182: "A probability of 1 ...", does this sentence refer to the percentage explained in the previous sentence or to the probability matrix B (defined later)? It is confusing to switch terminology between "percentage" and "probability".	4.6.<br>We understand it is confusing to switch terminology between "percentage" and "probability", and the term "percentage" appears not very appropriate in this situation. In order to avoid confusion we replaced "percentage" by "proportion" and we added a sentence explaining how we derived this "proportion" as a "probability" (Line 183 to 187)
	4.7.<br>Line 256: "the surface of the domain of separability" has no defintion but seems to refer to the proportion of matrix elements where the value is greater than 0.9 (based on caption of fig. 3). It would be good to explicitly define what "surface of the domain of separability" means here, where the term first appears.	4.7.<br>We filled this gap in the Materials and Methods section : *We arbitrarily consider that a probability larger than 0.9 allows discrimination between two models amongst the four that are used and allows us to define the domain of separability. We also defined the surface of the domain of separability as the proportion of cells in the matrix of probability where the value is larger than 0.9* (Line 192 to 195)
	4.8.<br>The final paragraph of the Conclusion section, from line 393 onwards, is worded strangely, and I'm not quite sure what it's trying to say. It would be good to clarify the language.	4.8.<br>We have reformulated this last paragraph (Line 403 to 410)
